# Exploring microbial functional biodiversity at the protein family level—From metagenomic sequence reads to annotated protein clusters

**DOI:** 10.3389/fbinf.2023.1157956

**Published:** 2023-03-03

**Authors:** Fotis A. Baltoumas, Evangelos Karatzas, David Paez-Espino, Nefeli K. Venetsianou, Eleni Aplakidou, Anastasis Oulas, Robert D. Finn, Sergey Ovchinnikov, Evangelos Pafilis, Nikos C. Kyrpides, Georgios A. Pavlopoulos

**Affiliations:** ^1^ Institute for Fundamental Biomedical Research, BSRC “Alexander Fleming”, Vari, Greece; ^2^ Lawrence Berkeley National Laboratory, DOE Joint Genome Institute, Berkeley, CA, United States; ^3^ The Cyprus Institute of Neurology and Genetics, Nicosia, Cyprus; ^4^ European Molecular Biology Laboratory, European Bioinformatics Institute (EMBL-EBI), Wellcome Genome Campus, Cambridge, United Kingdom; ^5^ John Harvard Distinguished Science Fellowship Program, Harvard University, Cambridge, MA, United States; ^6^ Institute of Marine Biology, Biotechnology and Aquaculture (IMBBC), Hellenic Centre for Marine Research (HCMR), Heraklion, Greece; ^7^ Center of New Biotechnologies and Precision Medicine, Department of Medicine, School of Health Sciences, National and Kapodistrian University of Athens, Athens, Greece; ^8^ Hellenic Army Academy, Vari, Greece

**Keywords:** protein clustering, metagenomes, metatranscriptomes, cluster annotation, biodiversity, microbial dark matter, protein families

## Abstract

Metagenomics has enabled accessing the genetic repertoire of natural microbial communities. Metagenome shotgun sequencing has become the method of choice for studying and classifying microorganisms from various environments. To this end, several methods have been developed to process and analyze the sequence data from raw reads to end-products such as predicted protein sequences or families. In this article, we provide a thorough review to simplify such processes and discuss the alternative methodologies that can be followed in order to explore biodiversity at the protein family level. We provide details for analysis tools and we comment on their scalability as well as their advantages and disadvantages. Finally, we report the available data repositories and recommend various approaches for protein family annotation related to phylogenetic distribution, structure prediction and metadata enrichment.

## 1 Introduction

Microbes are the most abundant and diverse life forms on the planet, occupying all possible metabolic niches. Cellular organisms such as bacteria, archaea and protista, as well as non-cellular entities such as viruses, can be found in all types of diverse ecosystems, from soils, rivers and oceans to extreme environments such as deserts, hot springs and glaciers, or as parasites in multicellular organisms such as humans and mammals, fish, insects and plants (Keller and Zengler, 2004; Thompson et al., 2017; Seshadri et al., 2018; Nayfach et al., 2021). The number of microorganisms surpasses by far the number of all other life forms; in fact, it is estimated that the number of microbes in a handful of soil exceeds the number of stars in the Milky Way galaxy (Whitman et al., 1998; Mukherjee et al., 2017). Microorganism communities, also known as microbiomes, play crucial roles in all ecosystems, from regulating carbon fixation and nutrient cycles to influencing the health, physiology, behavior, and ecology of their host organisms. As a result, the study of microorganisms and microbial communities is crucial, with applications in biomedicine, biotechnology, ecology and the study of biodiversity. Despite their importance, the vast majority of microorganisms and their genetic contents remain unannotated. The genomes of less than half a million microbes have been sequenced (Mukherjee et al., 2022), and only ∼30,000 bacterial and archaeal species have been cultivated (Parte et al., 2020), representing less than 1% of the total number of microbial taxa on Earth. Instead, the vast majority of microbial life remains taxonomically and functionally unknown (Locey and Lennon, 2016), often referred to as the “microbial dark matter”.

A central approach in exploring the functional diversity of the microbiome is through metagenomics, defined as the total amount of sequenced genetic material from an environmental sample (Oulas et al., 2015). Metagenomic shotgun sequencing has emerged as the most prevalent way of studying and classifying microorganisms from various habitats (Escobar-Zepeda et al., 2015; Quince et al., 2017; Liu et al., 2021). The latest advances in high-throughput shotgun sequencing technologies have improved the quality and reduced the cost of the method, resulting in a very large increase in the volume of available metagenomic sequences, which provide a great resource for new findings and novelty (Oulas et al., 2015; Pérez-Cobas et al., 2020).

**FIGURE 1:**
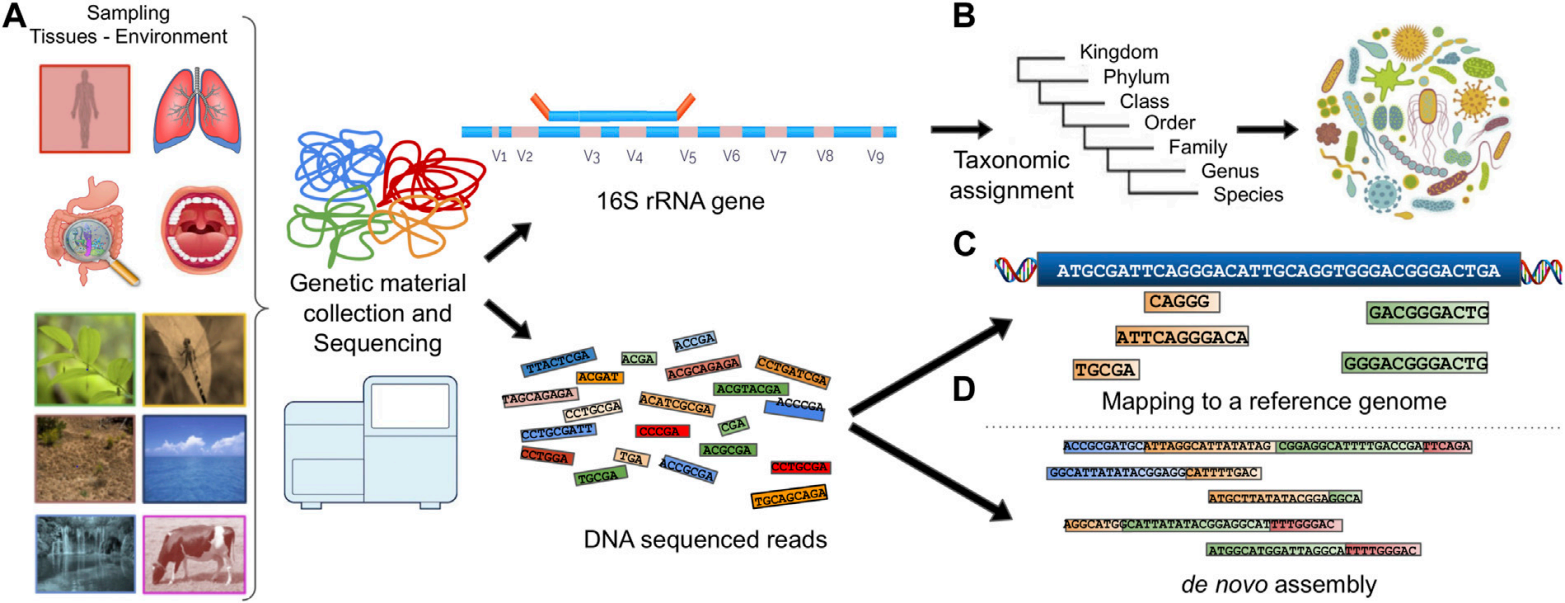
Illustration of a typical metagenomic analysis. **(A)** Sample collection, **(B)** Marker gene detection and taxonomic assignment. **(C)** DNA reads are mapped to a reference genome. **(D)** DNA reads are assembled into contigs using *de novo* assembly.

Extracting the genetic composition in a metagenomic sample usually follows one of the following paths (Figure 1):

- The genetic material is processed for marker gene detection (Rotimi et al., 2018) or characteristic genomic regions (e.g., 16S and 18S (Karst et al., 2018), Internal Transcribed Spacers (ITS), COI based on the SILVA (Pruesse et al., 2007; Porter and Hajibabaei, 2020), UNITE (Nilsson et al., 2019), PR^2^ (Del Campo et al., 2018) and MIDORI (Leray et al., 2018) database information respectively). This method can be used to describe the microbial composition based on the taxonomic groups present in the sample and is frequently used to analyze the biodiversity of microbial ecosystems.
- The reads produced by a sequencer can be accurately mapped to multiple known and annotated reference genomes or metatranscriptomes, providing information about genes, proteins and the available functions thereof.
- In the case of zero matches to a reference genome, the reads are assembled into *contiguous sequences* known as *contigs* which are sets of overlapping DNA segments that together represent a consensus DNA region. The contigs can be further assembled into sets with gaps of known lengths, forming *scaffolds.* This process is called *de novo assembly.*

Once reads have been aligned to a reference genome, functional annotation can be straightforward if the reference genome is well annotated and one can identify the functions based on the genomic regions to which the reads are aligned. On the other hand, functional annotation of assembled scaffolds, e.g., open reading frame calling or protein function prediction, can be tricky, as reference information is often limited or unavailable. Clustering predicted proteins into groups (families) can both shed light on putative protein functions and, more practically, reduce the number of proteins present in metagenomic datasets into more manageable chunks.

Going beyond the available literature, in this review we provide a step-by-step methodology on how to explore diversity at the protein family level with the use of metagenomic data. We discuss the available data repositories and their contents, pipelines related to read mapping, assembly and end-product (e.g., protein sequences) generation, as well as graph-based and non-graph-based clustering techniques (Zaslavsky et al., 2016; Pavlopoulos, 2017). Finally, we recommend ways to annotate the protein clusters with information on function, environment, and geography.

## 2 Data repositories

The analyzed metagenomic and metatranscriptomic data and metadata, including their datasets, sequencing scaffolds, predicted genes and annotations, are hosted in a number of publicly available databases and repositories. This section presents the most important hubs of metagenomic data, including their data contents and offered metagenome analysis services.

The Integrated Microbial Genomes and Microbiomes (IMG/M) database (Chen et al., 2018; Chen et al., 2022) is a user-driven repository hosted by the Joint Genome Institute (JGI) of the US Department of Energy (DOE) (Chen et al., 2018; Chen et al., 2022). It includes genomes of cultivated and uncultivated taxa from all domains of life (Archaea, Bacteria, Eukarya and Viruses), plasmids, genome fragments of interest generated by targeted sequencing, amplicons, metagenomes and metatranscriptomes. In its current version (v. 7.0 February 2023 data), the database contains 172,782 datasets, 47,113 of which are metagenomic (39,610 metagenomes and 7,503 metatranscriptomes). IMG/M’s datasets contain 23.29 trillion base pairs, 11.94 trillion of which are protein-coding and correspond to 70.18 billion protein sequences. Metagenomes and metatranscriptomes are the main contributors to these figures, containing 22.81 trillion base pairs (11.55 trillion protein-coding) that encode 69.77 billion proteins. While a portion of these sequences are retrieved from other repositories, namely, GenBank (Sayers et al., 2022) and the Sequence Read Archive (SRA) (Kodama et al., 2012), the majority of IMG/M’s content comes from datasets sequenced at the JGI itself, as well as datasets submitted by external users through the IMG submission system. The database features a well-established, continuously updated metagenome analysis pipeline (DOE JGI Metagenome workflow), allowing users to submit their own genome, metagenome and metatranscriptome datasets, and automatically perform several types of analyses, including gene calling, taxonomic assignment and functional annotation (Clum et al., 2021).

Similar to IMG/M, MGnify, previously known as EBI Metagenomics (Mitchell et al., 2018), is a freely available database for the archiving, exploring and analyzing metagenomic data, hosted by the European Bioinformatics Institute (EBI) (Mitchell et al., 2019). The database accepts user-submitted data and provides a versatile, standardized pipeline (EBI metagenomics pipeline) to cover the analysis of a wide range of dataset types, from studies targeting taxonomic markers (e.g., amplicon studies) to shotgun sequencing of metagenomes and metatranscriptomes, as well as metagenome-assembled genomes (MAGs). The pipeline offers various types of analyses (gene calling, functional annotation, taxonomic assignment) for user-submitted assembled sequence data, as well as the option to provide assembly for user-submitted, raw reads upon request. In its current version (February 2023 data), MGnify hosts 444,172 analysis datasets coming from 4,444 studies, including, among others, 33,827 metagenomes, 2,205 metatranscriptomes, and 301,808 MAGs from seven major MAG catalogs. The aforementioned datasets encode a total of ∼2.5 billion protein sequences, grouped into ∼620 million clusters with a 90% sequence identity threshold. All sequence data deposited in MGnify are automatically submitted to the European Nucleotide Archive (ENA) catalog, in compliance with the International Nucleotide Sequence Database Collaboration (INSDC) standards (Cummins et al., 2022). Notably, MGnify hosts data from seven super studies, organized by large microbiome research groups and consortia. These include the Tara Oceans (Sunagawa et al., 2015), Malaspina 2010 and AtlantECO projects (collecting microbiome data from ocean expeditions), the Earth Microbiome Project (an effort to organize microbiome datasets from around the globe) (Thompson et al., 2017), Project MANGO from the NASA GeneLab database (collecting data on how microbial communities adapt to spaceflight and related terrestrial stresses) (Berrios et al., 2021), HoloFood (microbiome data from farmed animals and food production systems) and FindingPheno (studying the impact of host-microbiome interactions).

Besides IMG/M and MGnify, two other notable metagenome repositories are MG-RAST (Meyer et al., 2019) and gcMeta (Shi et al., 2019). The Metagenomes RAST service (MG-RAST), maintained by the Argonne National Laboratory at the University of Chicago, is one of the earliest approaches to providing an integrated platform for the automated analysis and annotation of metagenomic samples (Meyer et al., 2019). In contrast to IMG/M and MGnify, which operate as publicly available databases offering analysis pipelines alongside their data, MG-RAST acts primarily as a metagenome annotation pipeline, with access to its database restricted to its registered users. In addition, MG-RAST is limited to analyzing user-submitted metagenome reads and mapping them to reference genomes, rather than also analyzing full genomes, amplicons, assembled contigs/scaffolds or MAGs. In its current version (v. 4.0.3 February 2023 data) MG-RAST hosts 510,609 metagenomes, containing 2,266 billion sequences; however, only ∼16% of these (81,196 datasets) are publicly available to researchers. In contrast to MG-RAST, gcMeta (Shi et al., 2019) is a publicly available metagenome annotation platform and associated database, maintained by the Chinese Academy of Sciences Initiative of Microbiome (CAS-CMI). It utilizes a pipeline similar to IMG/M and MGnify in terms of sequence analysis and annotation, which primarily focuses on datasets submitted by members of CAS-CMI. In its current version (February 2023 data), gcMeta contains a total of 146,672 datasets, including 42,628 metagenomes, 1,431 metatranscriptomes, 3,980 genomes and 98,723 amplicons, that encode a total of 153,352 sequences. Although its data content is significantly smaller than that of MG-RAST, the majority of these datasets are publicly available, with only 2,305 studies held as private due to confidentiality restrictions.

Apart from the aforementioned major repositories, a number of smaller, more specialized databases have been made available, each focusing on different types of microbiome samples, or different approaches in metagenome analysis. A notable example is IMG/VR (Roux et al., 2021; Camargo et al., 2022), a subset of IMG/M focusing exclusively on viral genomes and metagenomes (Paez-Espino et al., 2017a). IMG/VR uses the DOE JGI Metagenome workflow to analyze its samples, coupled with additional analysis and annotation tools taking into account specialized aspects of viral samples, such as gene structure. Other databases host metagenomic samples based on their source ecosystems or biome types. TerrestrialMetagenomeDB (Corrêa et al., 2019), MarineMetagenomeDB (Nata’ala et al., 2022) and HumanMetagenomeDB (Kasmanas et al., 2021), hosted by the Helmholtz Center for Environmental Research, annotate SRA and MG-RAST metagenomes obtained from soil, marine and human microbiome samples, respectively. The Marine Metagenomics Portal (MMP) also holds and annotates a number of marine-oriented metagenomic datasets (Klemetsen et al., 2018), obtained from MGnify. Finally, the NIH Human Microbiome Project (Lloyd-Price et al., 2017) and MetaGeneBank (Shao et al., 2021) are two repositories focusing on metagenomes from human host-associated systems, such as the lung and gut microbiota. Notably, the majority of these resources do not contain directly submitted data; instead, they provide additional annotation and analysis for publicly available datasets coming from major resources such as IMG/M, MG-RAST or MGnify.

In addition to metagenome-focused databases, described above, metagenomic data have also been compiled into datasets containing clustered sets of metagenomic sequences, either DNA or proteins, usually at varying levels of sequence identity. One of the earliest examples in this category was UniMES (ANNOTATING UniProt METAGENOMIC AND ENVIRONMENTAL SEQUENCES IN UniMES, 2011), a metagenomic protein sequence repository that was maintained by UniProt. UniMES’s sequences were primarily collected from the Global Ocean Sampling (GOS) expedition and included translated protein sequences from more than 26 million microbiome samples. The repository was eventually retired in favor of MGnify; however, its sequences have been integrated into the UniParc archive, a non-redundant database that contains most of the publicly available protein sequences in the world. Another related sequence repository is hosted by the Tara Oceans expedition in collaboration with the European Molecular Biology Laboratory (EMBL) (Sunagawa et al., 2015), containing sequence sets clustered with CD-HIT (Li and Godzik, 2006). However, the most comprehensive set of clustered sequences in metagenomics is currently metaClust, a collection of more than 1.5 billion metagenomic protein sequences, clustered using MMseqs2 (Steinegger and Söding, 2018). The metaClust set contains sequences from IMG/M, MGnify, the Tara Oceans repository and UniParc, organized at various levels of redundancy.

The sheer volume of the data hosted by the database and repositories described above demonstrates the level of metagenomic contributions in the DNA and protein sequence space. In IMG/M alone, roughly 47,000 metagenomes and metatranscriptomes correspond to ∼23 trillion base pairs (bps) and ∼61.7 billion contigs, amounting to dozens of petabytes of data; by comparison, the equivalent measurements from IMG’s reference (isolate) genomes (IMG-NR) report only ∼478 billion bps and 12.4 million contigs. At the protein level, metagenome-derived protein sequences constitute 99.4% (69.77 billion sequences) of the repository’s content, exceeding the equivalent sequences from isolate genomes (∼413 million) by multiple orders of magnitude. A similar trend is observed in MGnify, despite the vast differences in the amount of data between the latter and IMG. For reference purposes, the combined non-metagenomic datasets of the INSDC [GenBank (Sayers et al., 2022), ENA (Cummins et al., 2022) and DDBJ (Okido et al., 2022)] constitute less than 2 billion entries (assembled sequences), while the UniParc archive contains 542.15 million protein sequences, only a fraction of which come from metagenomes. These numbers are further reduced when taking sequence annotation into account. In its current release (2022_05, retrieved February 2023), UniProtKB contains a total of 230, 149, 489 sequences (568,744 manually annotated entries in SwissProt and 229, 580, 745 computationally annotated entries in TrEMBL) (The UniProt Consortium et al., 2021). InterPro, a collection of protein classification databases based on sequence similarity that includes, among others, Pfam (Mistry et al., 2021), CATH-Gene3D (Sillitoe et al., 2021), PROSITE (Sigrist et al., 2013) *etc.*, hosts approximately 38,349 families (clusters), describing ∼193.6 million sequences (February 2023 data). Finally, the Clusters of Orthologous Genes (COG) database (Galperin et al., 2021) contains 4,877 functional classes for roughly 3.2 million protein sequences.

This tremendous discrepancy between sequences derived from standard methods and metagenomic sequencing showcases the importance of metagenomes in unveiling the functional dark matter. It also clearly highlights the need for developing highly scalable and parallelizable methods for parsing and analyzing such enormous volumes of data.

## 3 Metagenomic analysis and workflows

### 3.1 Assembly—Mapping and binning

Metagenomics studies are widely applied to investigate both known and novel genomes that exist within an environmental sample. To analyze such a sample, shorter reads are assembled into genomic contigs through the mapping process and subsequently into scaffolds to better understand the investigated organisms. During read mapping, reads are aligned to reference genomes from known organisms. This can be used to profile taxa present in the metagenomic samples, or to quantify the gene expression levels in metatranscriptomes. A short presentation of the approaches utilized in metagenome assemblies are given in this section. A more detailed description can be found in the review by Sedlazeck et al. (2018).

Before reads are assembled, a preprocessing analysis step is required. The specifics of this analysis heavily depend on the methods used for sequencing, and no consensus exists that can fully cover all different sequencing approaches. However, this step generally involves merging paired reads and performing a quality control (QC) analysis. These tasks are usually conducted using standard sequencing analysis tools. Merging can be conducted with dedicated, commercially available tools such as Real Time Analysis (RTA) from Illumina’s NovaSeq, or with open-source solutions such as SeqPrep and BBmerge (Bushnell et al., 2017). QC analysis can be performed using dedicated tools like FastQC and the FastX toolkit or, alternatively, with in-house scripts using popular programming languages such as Python (Biopython) (Cock et al., 2009) or R (Bioconductor) (Gentleman et al., 2004). Another notable example of a metagenome-focused QC analysis method is DRISEE, designed to detect high or varying levels of sequencing errors that may confound downstream analyses (Keegan et al., 2012). Based on the QC results, the analyzed reads may need to undergo a number of refinements, including the detection and removal of adapter sequences and the trimming of low-quality regions. Depending on the nature of the source samples, additional preprocessing may also be required, such as masking reads that can be mapped to host organisms (e.g., human) or known contaminants with a significant degree of sequence similarity (>93% identity) (Clum et al., 2021), or detecting and removing low complexity regions. Popular trimming tools include Skewer (Jiang et al., 2014) or Trimmomatic (Bolger et al., 2014), while low complexity regions can be detected and removed with tools such as DUST (Morgulis et al., 2006), Tantan (Frith, 2011) or TRhist (Doi et al., 2014). These tools can be used on their own, or in combination with additional methods through the data submission pipelines of repositories such as IMG/M or MGnify.

Following quality control, the reads can then be mapped to a reference genome, *de novo* assembled into scaffolds, or, if enough content is available, assembled into MAGs. Mapping to reference genomes can be performed using a wide range of different approaches. Notable examples for short read mapping include Stampy (Lunter and Goodson, 2011), Bowtie (Langmead and Salzberg, 2012), SOAP3 (Liu et al., 2012), MAQ (Li et al., 2008) and MOM (Eaves and Gao, 2009). For longer read mapping, BWA-SWA/BWA-MEM (Houtgast et al., 2018) and Bowtie 2 (Langmead and Salzberg, 2012) are currently the most widely used choices. Other mapping methods include MicroRazerS (Emde et al., 2010), which specializes in aligning short RNA-seq reads, X-mate, an integrated pipeline capable of aligning both DNA and RNA-seq datasets (Wood et al., 2011) and BBtools (Bushnell et al., 2017), which is a collection of tools, currently used by the IMG/M database, that was designed for handling paired-end shotgun reads from high-throughput sequencing platforms. Reference genomes can be accessed through databases such as NCBI RefSeq (Li et al., 2021), UCSC (Tyner et al., 2017), Ensembl (Zerbino et al., 2018) and the International Genome Sample Resource (IGSR) (Fairley et al., 2020).

Binning is the process of grouping reads or contigs into individual genomes and assigning each group to a specific species, subspecies, or genus, where possible. An environmental sample may contain reads or contigs originating from many different microorganisms. By grouping the reads into bins that characterize unique taxonomic lineages, the assembly process is better facilitated and allows for more accurate contigs to be generated. Established binning tools are discussed in-depth elsewhere (Wang et al., 2017). Some of these tools include: MetaBAT2 (Kang et al., 2019), GroopM (Imelfort et al., 2014), MaxBin 2.0 (Wu et al., 2016), COCACOLA (Lu et al., 2016), CONCOCT (Alneberg et al., 2013), Autometa (Miller et al., 2019), MetaWatt (Strous et al., 2012), SCIMM (Kelley and Salzberg, 2010), Metacluster 5.0 (Wang et al., 2012), LikelyBin (Kislyuk et al., 2009), AbundanceBin (Wu and Ye, 2011), SolidBin (Wang Z. et al., 2019), Vamb (Nissen et al., 2018), Binsanity (Graham et al., 2017), BMC3C (Yu et al., 2018) and MyCC (Lin and Liao, 2016). The review of Mande et al. (2012) also provides more in-depth information regarding binning methodologies and their advantages and limitations. In a recent paper (Yue et al., 2020), 15 binning tools were compared on a chicken gut metagenome dataset. In general, MetaBat, Groopm2 and Autometa outperformed the rest of the tools (Borderes et al., 2021).

Following the binning process, contigs can be further assembled into scaffolds. Assembling a genome *de novo* from contigs and scaffolds, by utilizing paired-end reads to avoid repetitions, produces MAGs (Lapidus and Korobeynikov, 2021). Tools that are used for metagenomic assembly are divided into two groups, utilizing either short- or long-read sequences respectively (Yang C. et al., 2021). Short-read metagenomic assembly software includes tools such as metaSPAdes (Nurk et al., 2017), MetaviralSPAdes (a variant of the former for viral metagenomes) (Antipov et al., 2020), Plass (Steinegger et al., 2019b), MEGAHIT (Li et al., 2015), MetaVelvet (Namiki et al., 2012), Omega (Haider et al., 2014), Ray Meta (Boisvert et al., 2012) and IDBA-UD (Peng et al., 2012). Long-read assemblers include Athena (Bishara et al., 2018), cloudSPAdes (Tolstoganov et al., 2019), Nanoscope (Kuleshov et al., 2016), Canu (Koren et al., 2017), NECAT (Chen et al., 2021), wtdbg2 (Ruan and Li, 2020) and metaFlye (Kolmogorov et al., 2020). Similarly to standard reference genomes, MAGs are also deposited into dedicated repositories. Some established MAG catalogs include the Genomes from Earth’s Microbiomes (GEM) catalog (Nayfach et al., 2021) (∼*52K* MAGs - where all public MAGs are also uploaded in GenBank (Benson et al., 2018)); the European Nucleotide Archive (ENA) (*∼37K* MAGs) (Cummins et al., 2022); MGnify (*∼10K* genomes in four MAG catalogs) (Mitchell et al., 2019), which is both a MAG resource as well as an analysis pipeline for MAGs from ENA; the OceanDNA MAG catalog, which contains *52,325* prokaryotic MAGs from marine environments submitted to the DNA Data Bank of Japan (DDBJ) (Mashima et al., 2016); and the integrated mouse gut metagenome catalog (iMGMC) (*660* MAGs) (Lesker et al., 2020).

### 3.2 Gene calling and annotation

Following the successful assembly of the sample reads, the next step is annotation. This stage involves identifying genes (both protein-coding and non-protein coding) and other sequence or genomic structure features [e.g., CRISPR arrays (Mohamadi et al., 2020)], and providing each feature with a meaningful list of hints about its possible biological function. However, what sets annotation apart from other computational steps in processing metagenomic data is that no reliable benchmarks for annotation tools exist (Dong and Strous, 2019). Thus, choosing an appropriate workflow depends on the nature of the data, the available computational resources and the researcher’s background and preferences in analysis methods. In theory, metagenomic data can be analyzed with any combination of sequence analysis tools. In practice, the most employed methods for annotation usually come in the form of automated pipelines, either standalone or integrated into databases, and other online services. Notable online examples include the DOE JGI Metagenome workflow (Clum et al., 2021) (used by IMG/M and other associated resources), EBI Metagenomics (Mitchell et al., 2019) (used by MGnify), MG-RAST (Meyer et al., 2019), MicroScope (Vallenet et al., 2017) and MetaErg (Dong and Strous, 2019). Commonly used standalone packages are the NCBI Prokaryotic Genome Annotation Pipeline (PGAP) (Tatusova et al., 2016), Prokka (Seemann, 2014) and DFAST (Tanizawa et al., 2018).

**FIGURE 2:**
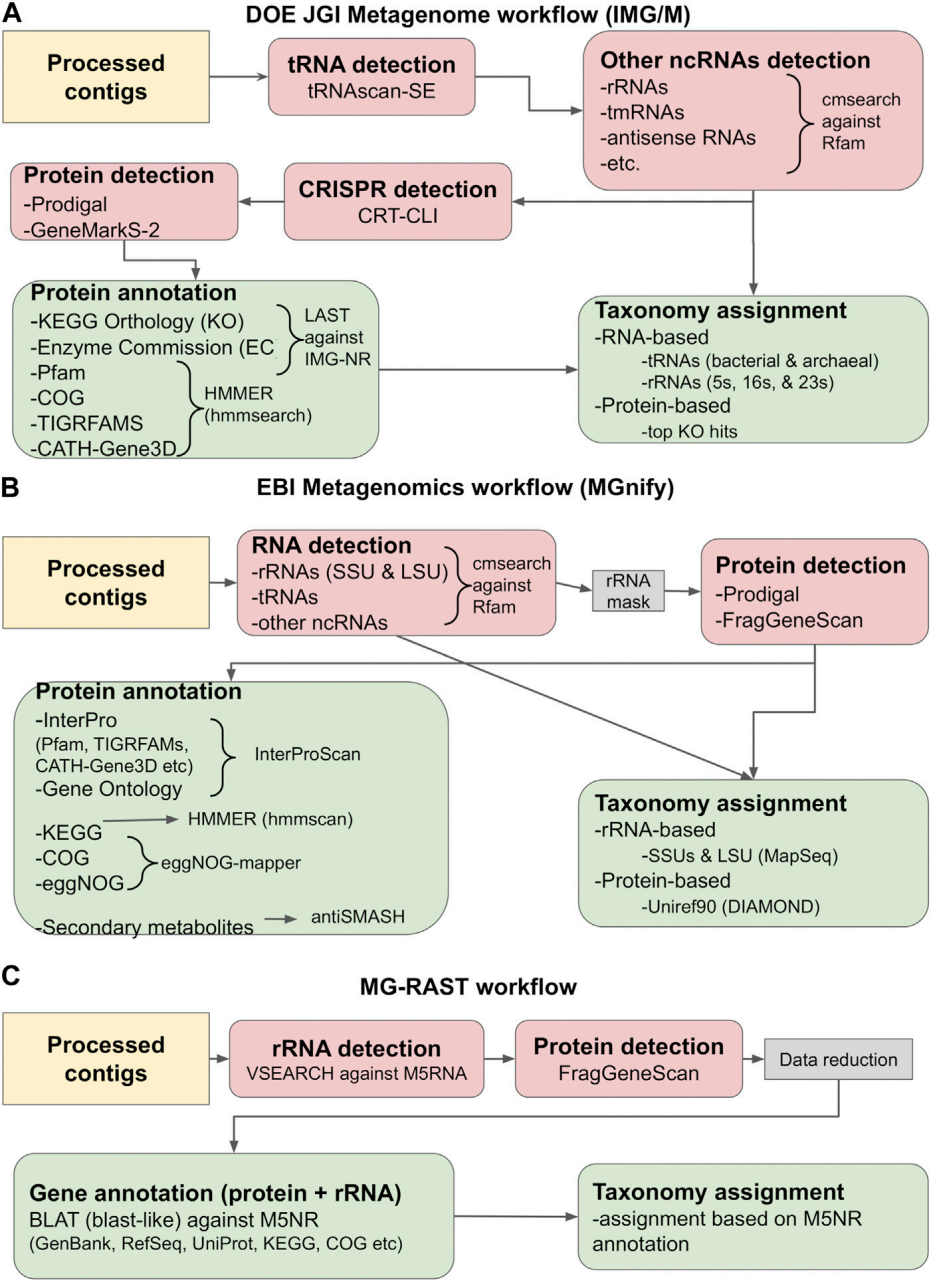
Gene calling and annotation in IMG/M **(A)**, MGnify **(B)** and MG-RAST **(C)**. Simplified overviews of the three workflows are shown. Gene calling operations (RNA or protein) are colored salmon pink, while gene annotation operations are colored light green. The tools used in each workflow are given in the graph and described in the main text. The workflows are based on the methodology described in Clum et al. (2021), Mitchell et al. (2019) and Meyer et al. (2019).

For the purposes of this review, we will focus on the methods and tools employed by the three most commonly used metagenome repositories: the DOE JGI Metagenome (IMG/M), EBI Metagenomics (MGnify) and MG-RAST pipelines. A simplified view of their annotation workflows is given in Figure 2. The procedures followed are presented from the scope of analyzing assembled contigs; however, the pipelines also support the annotation of amplicons, fragments, and, in the case of MGnify, unassembled reads, by using most of the same tools. Some specific details differentiate among the workflows, as each may use different tools for the same type of annotation, or perform additional analyses; for example, the DOE JGI pipeline also searches for CRISPR elements (Anzalone et al., 2020; Makarova et al., 2020; Nidhi et al., 2021; Chavez et al., 2022; Katti et al., 2022; Wang et al., 2022) with CRT-CLI (Bland et al., 2007; Clum et al., 2021). However, all three workflows follow, more or less, the same procedure, which consists of the following stages: *i)* the detection of non-coding RNA (ncRNA) genes, *ii)* the prediction of protein-coding genes, and *iii)* functional annotation of proteins and taxonomic assignment.

The first step in annotating the assembled reads is detecting non-coding RNAs (ncRNAs). These primarily include ribosomal RNAs (rRNAs), transfer RNAs (tRNAs) and other categories such as antisense RNAs, transfer-messenger RNAs (tmRNAs), *etc.* Detecting ncRNAs can provide an initial taxonomic annotation of the assembled reads that can then be used to correctly identify protein-coding genes. In addition, identifying and masking the position of ncRNAs can help reduce the number of falsely translated protein sequences by discarding potential open reading frames (ORFs) that overlap with ncRNA coordinates. Detection is typically performed by running DNA or RNA sequence queries against one or more RNA family databases. The most prominent database in this category is Rfam (Kalvari et al., 2021), a manually curated collection of RNA families. In its current version (November 2022, retrieved February 2023), Rfam contains 4,108 families, each represented by multiple sequence alignments, consensus secondary structures and Covariance Models (CMs). The latter are probabilistic models of the conserved sequence and secondary structure for an RNA family, analogous to the Hidden Markov Model (HMM) profiles commonly used for protein sequence analysis (Nawrocki and Eddy, 2013).

Currently, the most robust RNA detection method is INFERNAL (INFERence of RNA ALignment), which can perform DNA sequence searches against RNA reference databases using CM profiles (Nawrocki and Eddy, 2013). The *cmsearch* utility of INFERNAL and the Rfam database are used by both IMG/M and MGnify to detect non-coding RNAs in metagenome assemblies. The IMG/M workflow also uses tRNAscan-SE, a tool specifically designed to detect tRNAs using CMs and perform basic taxonomic assignment (Chan et al., 2021). Contrary to the above, MG-RAST performs sequence-based rRNA searches against M5RNA, a subset of the M5NR database (Wilke et al., 2012) containing non-redundant rRNA sequences, using VSEARCH (Rognes et al., 2016), an open-source alternative of the usearch tool (Edgar, 2010). Another useful tool is MapSeq (Matias Rodrigues et al., 2017), a *k*-mer based rRNA sequence search and analysis tool that is used by MGnify to analyze *cmsearch* results and provide SSU and LSU taxonomy assignment. Finally, the identified RNA genes can be used to establish a generalized functional profile for the analyzed sample, using functional annotations from reference genomes with matches to the detected marker regions. One notable tool performing this functionality is PICRUSt, designed for the functional profiling of microbial communities using 16S rRNA marker gene sequences (Langille et al., 2013).

Having identified the positions of ncRNA genes, the next step in the analysis is the prediction of protein-coding genes. Generally, this is performed by identifying and translating potential ORFs and selecting the highest confidence results. However, compared to standard genomics analysis, this particular step poses a number of challenges for metagenomes, many of which are directly related to the nature of the metagenomic data themselves. Since the source organism of a metagenomic sequence is typically not known, special care must be taken in selecting the proper genetic code for translating the sequence. Another problem arises from the GC content of the samples. Standard gene recognition methods perform relatively well in low GC-content genomes, but their accuracy drops considerably in high GC-content sequences. The latter contain fewer stop codons and more spurious ORFs, often resulting in false protein translations (Chen and Pachter, 2005). Finally, one important issue to address is metagenome fragmentation, which can lead to incomplete genes (fragments) and sequencing errors such as frameshifts, further complicating gene prediction. Early metagenomic studies addressed these issues by utilizing homology-based methods, i.e., searching the input sequences against reference databases with tools such as BLAST (Altschul et al., 1990). Notably, MG-RAST utilized this method in its initial version (Meyer et al., 2008). Still, homology-based methods cannot predict novel genes, even though their discovery is a key focus of metagenomics. For this reason, a number of specialized gene calling methods have been developed, based on various types of statistical models. Early examples of metagenome-related gene prediction tools included MetaGene (Noguchi et al., 2006) and MetaGeneAnnotator (Noguchi et al., 2008), which detected prokaryotic gene structure using self-training logistic regression models based on start/stop codon distance and GC content. Another example was GeneMark.hmm (Besemer and Borodovsky, 1999) and its successor, GeneMarkS (version 1) (Besemer, 2001), both of which used heuristic approaches. However, the accuracy of these methods has been found to significantly decrease as the sequencing error rate increases (Hoff, 2009; Zhu et al., 2010).

More recently, gene prediction methods have been developed that are based on machine learning. The most popular tools in this category are FragGeneScan (Rho et al., 2010), Prodigal (Hyatt et al., 2010) and GeneMarkS-2 (Lomsadze et al., 2018). FragGeneScan utilizes two-level representation Hidden Markov Models (HMMs) to detect and translate protein genes on both strands for short and error-prone sequencing reads. It operates by detecting the best path of hidden states that is most likely to generate the observed nucleotide sequence. FragGeneScan reports genes if they meet the following three conditions: *i)* the length of each gene is longer than 60 bp, *ii)* the genes start in a start state (start codon) or in a match state (internal region of genes) and *iii)* the genes end in a stop state (stop codon) or in a match state (internal region of genes). As such, it is particularly useful for detecting partial (fragmented) genes without start or stop codons, alongside complete sequences (Rho et al., 2010). Another popular tool is Prodigal, which is based on dynamic programming (Hyatt et al., 2010) and can be used both for complete genomes and for metagenomic sequences (Hyatt et al., 2012). Prodigal has been trained in an unsupervised fashion using reference genomes from the JGI ORNL pipeline, to recognize general features including start codon usage, ribosomal binding site motifs, GC bias and other information necessary to build a complete training profile. Based on these features, it assigns a preliminary coding score for each potential gene and performs multiple types of dynamic programming across the whole sequence to detect the most probable gene model (Hyatt et al., 2010). Finally, GeneMarkS-2, a re-implementation of GeneMarkS, uses a multiple iteration approach based on Markov chains that combines the original, typical prokaryotic model with 41 atypical bacterial and archaeal models (Lomsadze et al., 2018).

The final step in the analysis is functional annotation. This is largely performed by searching the predicted proteins against reference databases and identifying potentially homologous sequences. Sequence-based tools, such as BLAST (Altschul et al., 1990), BLAT (Kent, 2002), LAST (Edgar, 2010), MMseqs-2 (Steinegger and Söding, 2017) and DIAMOND (Buchfink et al., 2015), or HMM-based implementations, such as HMMER (*hmmsearch/hmmscan*) (Eddy, 2011) and HH-suite (*hhblits/hhsearch*), (Steinegger et al., 2019a), can perform searches against RefSeq (Li et al., 2021), IMG-NR (Chen et al., 2018), UniProt (UniProt Consortium, 2018), Uniref, M5NR (Wilke et al., 2012) and other reference sequence repositories. Structural and domain annotation can also be performed by searching protein family databases such as Pfam (Mistry et al., 2021), TIGRFAMS (Haft et al., 2003) and others with HMM-based searches. Notably, the InterPro database has evolved to include profiles for all major protein family databases (Pfam, TIGRFAMS, *etc.*), allowing the simultaneous search of the above with a single operation through InterProScan (Jones et al., 2014; Blum et al., 2021). Through the results of the aforementioned searches, the functions of metagenomic sequences can be further annotated by matching them to KEGG orthologs and pathways, COG and eggNOG categories, enzyme reactions, secondary metabolites or Gene Ontology terms with dedicated tools such as KEGG Mapper (Kanehisa and Sato, 2020), eggNOG-mapper (Cantalapiedra et al., 2021) or antiSMASH (Blin et al., 2021). Topological features can be annotated through the use of prediction algorithms, such as SignalP (Teufel et al., 2022) for signal peptides, and TMHMM (Krogh et al., 2001) or Phobius (Käll et al., 2007) for transmembrane segments. Finally, the top most significant results of sequence homology searches can be used alongside data obtained from ncRNA gene calling (rRNA, tRNA, *etc.*) to provide taxonomic assignment for the assembled contigs.

### 3.3 Taxonomy assignment and phylogenetic distribution

Characterizing a contig at different taxonomic levels (domain, kingdom, phylum, class, order, family, genus, and species) is a very important and, at the same time, challenging task. Proper identification of a contig’s taxonomy is crucial for establishing its phylogenetic distribution, elucidating the phylogenetic content of a metagenomic sample and, ultimately, establishing the sample’s microbial diversity. As it was described in the previous section, major annotation pipelines such as those used by IMG/M, MGnify and MG-RAST, can perform an initial taxonomic assignment during gene calling; this is typically performed by searching for marker RNA genes and, if applicable, by evaluating the identity of predicted protein sequence hits to reference datasets. However, this annotation is not always adequate, resulting in a generalized taxonomy assignment (e.g., to the level of kingdom, phylum or class), rather than specific assignment to an order, family, genus or species. At the same time, a lot of metagenomic contigs often lack ncRNA genes or other marker regions and remain unclassified by the annotation pipelines. As a result, more specialized approaches need to be used. In this section, we analyze the most commonly used taxonomy assignment and phylogenetic distribution methods, in order to get an in-depth understanding of the procedures used to determine a metagenome’s phylogenetic content, as well as the evolutionary connection between the different lineages.

Several tools have been implemented for the taxonomic annotation of metagenomic reads and contigs. Most of these methods rely on one of three approaches: machine learning, alignment-based mapping or *k*-mers identification. The Naive Bayes Classifier tool (NBC) is a Bayesian statistics-based machine learning implementation to classify genomes and contigs by analyzing sequence motif frequencies (Rosen et al., 2011). Another machine learning-based tool, PhymmBL, utilizes Interpolated Markov Models (IMMs), with Markov chains using a variable number of states to compute the probability of the next state. The IMMs of the tool can be used to classify sequences based on patterns of DNA unique to a clade, which can be a species, genus, or higher-level phylogenetic group (Brady and Salzberg, 2009). Other methods take advantage of high quality sequence alignment algorithms, such as Bowtie (Langmead and Salzberg, 2012), BWA (Li and Durbin, 2009), MMseqs-2 (Steinegger and Söding, 2017) and DIAMOND (Buchfink et al., 2015), to identify contig regions that match with bacterial, archaeal, eukaryotic, or viral sequences. Combining this information with the alignment coverage, these tools can then recommend a lineage classification. MGmapper (Petersen et al., 2017) is one notable pipeline in this category, utilizing BWA-mem for aligning sequencing reads to reference databases and keeping the results with the highest sum of alignment scores. A similar tool, MetaPhlAn, uses bowtie2 to taxonomically map metagenomic shotgun sequencing data against an extensive database of ∼5.1 million unique clade-specific marker genes, identified from ∼1 million microbial genomes (Segata et al., 2012; Truong et al., 2015, 2; Blanco-Miguez et al., 2022). Other approaches perform gene calling and map the produced predicted genes to reference datasets to infer taxonomy. A popular method with this implementation is Kaiju (Menzel et al., 2016), which translates all potential ORFs with a generalized model and maps the predicted sequences to a user-defined reference protein database with a Burrows-Wheeler algorithm. Another example is the CAT (Contig Annotation Tool) and BAT (Bin Annotation Tool) set of classifiers (von Meijenfeldt et al., 2019), which use Prodigal to perform gene calling and compare the results against the NCBI BLAST-*nr* database with DIAMOND. The MMseqs-2.0 package also contains a taxonomy assignment tool (*mmseqs taxonomy*) for metagenomic contigs that functions by extracting all possible protein fragments from each contig, retaining only those that can contribute to taxonomic annotation and assigning their taxonomic identity through weighted voting (Mirdita et al., 2021). Finally, *k*-mer methods classify by identifying subsequences or “words” of length *k* (*k-mers*) contained in the contig sequences that can serve as a species-unique signature. So far, *k*-mer based tools such as Kraken 2 (Wood et al., 2019) and Centrifuge (Kim et al., 2016) have been the most successful in taxonomically classifying bacterial contigs.

One important limitation of all aforementioned classification methods is that they were largely designed with prokaryotic (bacterial and archaeal) samples in mind. Alignment-based and *k*-mer-based methods are generally capable of assigning taxonomy to eukaryotic contigs, often up to the species level; however, their success depends on the existence of reference databases. Furthermore, some of these methods depend on accurate gene prediction, which, paradoxically, requires knowledge of at least the kingdom level to produce reliable results (Pronk and Medema, 2022). For this reason, a number of tools have been developed that try to distinguish between prokaryotes and eukaryotes in metagenomic scaffolds. A popular method in this category is EukRep, a *k*-mer-based Support Vector Machine (SVM) classifier trained on binned data, that can be used to annotate binned metagenomes (West et al., 2018). Another example is EukDetect, which uses bowtie2 to align reads to a specially designed, extensive eukaryotic reference database (Lind and Pollard, 2021). Tiara, a machine-learning approach trained to detect organelle sequences, is capable of distinguishing between bacterial, archaeal, mitochondrial and eukaryotic samples (Karlicki et al., 2021). Finally, Whokaryote is a random forest classifier that uses manually selected features based on fundamental differences in gene structure between eukaryotes and prokaryotes, such as intergenic distance, contig gene density and the existence of ribosome-binding motifs (Pronk and Medema, 2022).

Identifying viral sequences from metagenomic samples is another category that requires the use of specialized tools. Viral genome structures are markedly different from that of cellular genomes, and are very diverse (DNA or RNA-based, single- or double-stranded *etc.*) (Chaitanya, 2019). While some of the aforementioned taxonomy assignment methods, such as Kraken 2 or MetaPhlAn, can rapidly map reads to known viral reference genome databases, the latter are biased towards those that have been isolated in the lab, leaving out the vast majority of the viral diversity (Paez-Espino et al., 2016; Paez-Espino et al., 2019). For this reason, the identification and annotation of viral content in metagenomic samples requires the use of specialized predictors. Early efforts in the field utilized prophage and provirus identification tools, designed to detect inactive viral genomes that have been integrated into the genome of a host cell. Notable examples in this category include Phage_Finder (Fouts, 2006), Prophinder (Lima-Mendez et al., 2008), Prophage HUNTER (Song et al., 2019), and PHAST/PHASTER (Arndt et al., 2016). These predictors primarily operate by detecting microbial gene regions with hits to isolated viral sequences; meaning that their ability to detect free-living lytic viruses from uncharacterized samples is limited. More recently, a number of metagenome-focused viral taxonomy tools and pipelines have been implemented; these are capable of handling fragmented and larger-scale microbial genomic datasets, and detecting viral components beyond prophages or close matches to reference datasets. Most of these methods rely on a combination of gene content and genomic structural features to distinguish viral from microbial sequences. A notable example in this category is the Earth Virome workflow (Paez-Espino et al., 2017b), an automated pipeline for the accurate detection and grouping of viral sequences from microbiome samples. The pipeline uses an expanded and curated set of viral protein families as “bait” to identify viral sequences directly from metagenomic assemblies. Notably, the Earth Virome workflow is used by the IMG/VR database for the identification and annotation of viral contigs from metagenomic samples (Roux et al., 2021; Camargo et al., 2022). Other tools include viralVerify, a component of MetaviralSPAdes that uses HMM-based searches and the NBC classifier to characterize Prodigal gene predictions (Antipov et al., 2020); MARVEL, which uses a random forest machine learning approach (Amgarten et al., 2018); VIBRANT, a pipeline combining HMM profile searches with neural networks and a unique metric to detect both free and integrated viruses (Kieft et al., 2020); MetaPhinder, an alignment-based method oriented towards detecting bacteriophages in assembled contigs (Jurtz et al., 2016); PhiSpy, which uses both similarity and composition strategies (Akhter et al., 2012); VirSorter2 (Guo et al., 2021), which combines a collection of customized automatic classifiers to evaluate sequence hits to viral reference datasets; and VirFinder, a *k*-mer based machine learning approach for viral contig identification that entirely avoids gene-based similarity searches (Ren et al., 2017). The latter has been used as the basis for DeepVirFinder (Ren et al., 2020), a deep learning method that uses convolutional neural networks, capable of detecting viral signals in very short contigs (<5,000 bps). Other recently developed deep learning tools include 3CAC (Pu and Shamir, 2022), a combined predictor of phages and bacterial plasmids, the bacteriophage-specific INHERIT (Bai et al., 2022), virSearcher (Liu et al., 2022), PHAMB (Johansen et al., 2022), Seeker (Auslander et al., 2020) and PhaMer (Shang et al., 2022) predictors and DeepMicrobeFinder (Hou et al., 2021), which classifies metagenomic contigs into five sequence classes (prokaryotic genomes, eukaryotic genomes, plasmids, prokaryotic-infecting viruses and eukaryotic-infecting viruses) with a reported accuracy of over 90% for viral contigs.

## 4 Sequence clustering strategies

Sequence clustering is the process of grouping biological sequences based on their similarity. The produced clusters can represent gene or protein families, containing members that are highly related to each other in terms of sequence identity and, therefore, may likely perform the same biological function. The above can be especially crucial in the study of metagenomes. Large-scale clustering can help reduce the large volume of metagenomic sequence data (as described in Section 2 of this review), by organizing sequences into groups and generating non-redundant sequence datasets and databases. At the same time, the produced clusters can be used to perform phylogenetic analysis and infer the evolutionary history and relationships of their members. Finally, clustering can be used as the basis for the functional annotation for previously unknown sequences, further reducing the metagenomic dark matter, either based on their coexistence in the same family as known genes and proteins or through the use of clusters in more advanced applications such as structure prediction. In this section of the review, we present three distinct approaches to sequence clustering, each with its own strengths and weaknesses, namely, sequence-based (also known as *k*-mer based), graph-based and hierarchical clustering.

### 4.1 Sequence-based clustering

Traditional applications such as BLAST (Altschul et al., 1990) or LAST (Edgar, 2010) enable querying a set of sequences against a protein database and subsequently allowing pairwise sequence comparisons where the query and target sequences alternate. However, the scalability of these applications is limited when millions of sequences must be processed.

**FIGURE 3:**
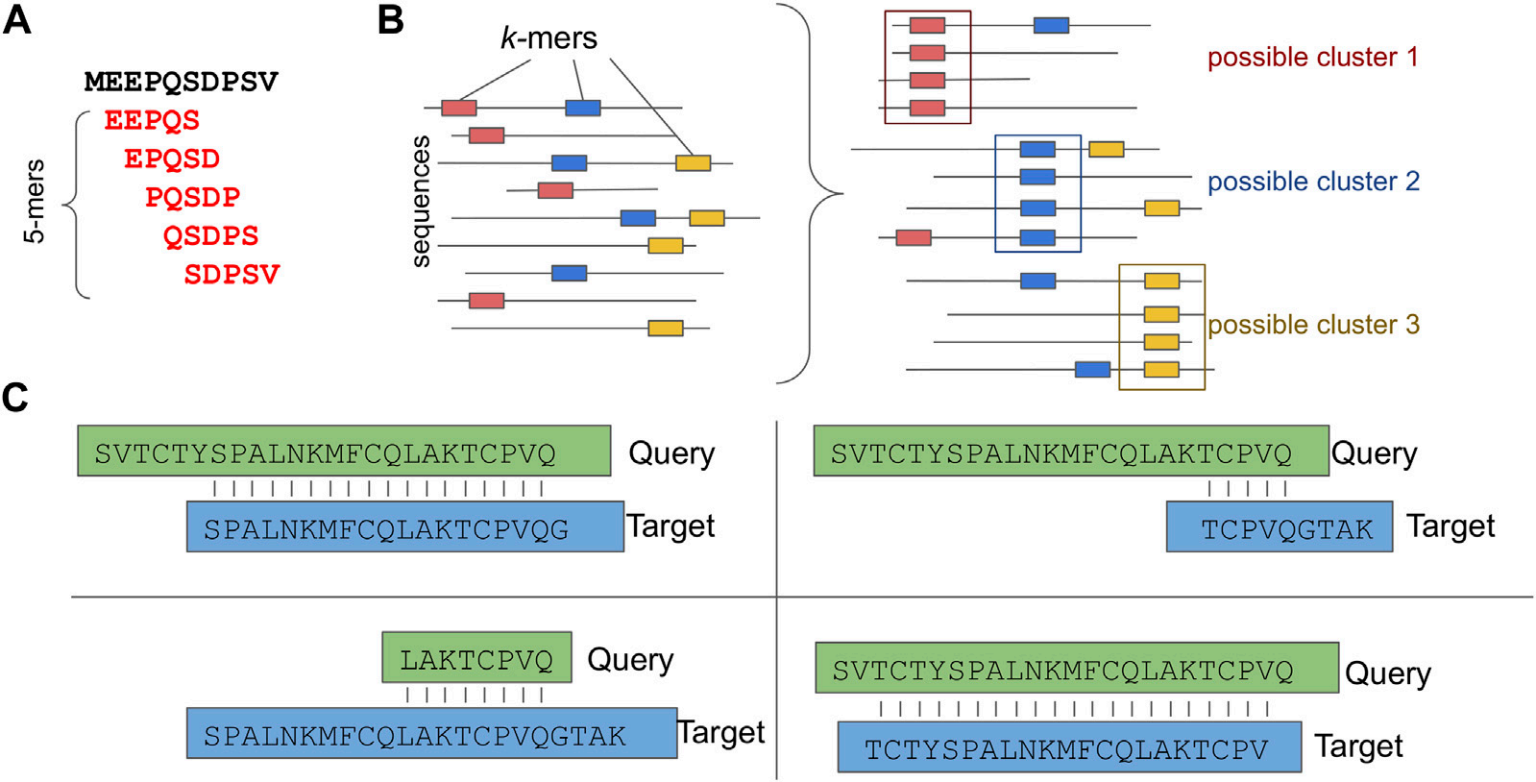
Sequence based Clustering. **(A)** A *k-*mer example, **(B)** Possible clusters based on common *k*-mers. **(C)** Different types of sequence assignment to clusters based on the alignment length coverage.

For this purpose, several sequence-based clustering applications that efficiently overcome the all-against-all comparison bottleneck have been introduced. Characteristic examples of such applications are: CD-HIT (Li and Godzik, 2006), DIAMOND (Buchfink et al., 2015), uclust/usearch (Edgar, 2010) and MMseqs2.0 (Steinegger and Söding, 2017). While each of these follows a unique clustering and sequence comparison approach, most of them allow sequence comparisons only for sequences that share common *k*-mers, thus skipping unnecessary calculations (Figures 3A, B). Notably, a *k*-mer is a substring of length *k* contained within a biological sequence.

Out of numerous available methods, MMseqs2.0 seems to be gaining ground and has been integrated into many pipelines of widely used databases [e.g., UniProt, UniParc (UniProt Consortium, 2018), MGnify (Mitchell et al., 2019)]. It uses MPI and OpenMP to run on multiple-CPU shared memory systems and uses a clustering methodology that is exhaustive, and thus time-consuming, but that also incorporates a heuristic approach, making it time-efficient [linclust (Steinegger and Söding, 2018)].

While the usability of most approaches is straightforward, taking into account the alignment length coverage percentage is of great importance when more uniform clusters are required. For example, Figure 3C depicts four different types of alignment: *a*) only sequences that have a sequence length overlap greater than x% of the longer of the two sequences are clustered; *b*) only sequences that have a sequence length overlap greater than x% of the target sequence are clustered; *c*) only sequences that have a sequence length overlap greater than x% of the query sequence are clustered and *d*) only sequences that have an alignment length overlap greater than x% bidirectionally are clustered.

Finally, a great advantage of MMseqs2.0 compared to its competitors is that new sequences can either be assigned to existing clusters (enrichment) or form new clusters without having to rerun the clustering from scratch. This is great for maintenance purposes when one wants to keep a database of sequence clusters up-to-date.

### 4.2 Graph-based clustering

**FIGURE 4:**
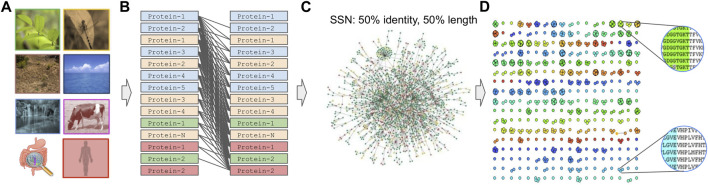
Graph-based family generation. **(A)** Sample collection, **(B)** All-against-all comparison. **(C)** SSN creation after applying, for example, an edge threshold of 50% identity, 50% alignment length. **(D)** Graph-based clustering.

Prior to graph clustering (Pavlopoulos et al., 2011; Koutrouli et al., 2020b), an all-versus-all sequence comparison is required to construct a sequence similarity network (SSN) (Figure 4). In such a network, nodes represent proteins or genes while edges represent the similarity between two amino acid or nucleotide sequences. Tools used for such comparisons are BLAST (Altschul et al., 1990), Last (Edgar, 2010), MMseqs-2.0 (Steinegger and Söding, 2017), PASTIS (Selvitopi et al., 2020; 2022) or dynamic programming approaches (Needleman and Wunsch, 1970). While the latter, along with BLAST, are the most exhaustive approaches, using them for large datasets is discouraging. On the contrary, LAST application is orders of magnitude faster than BLAST and, in the best case, one could process large datasets in parallel after splitting them into chunks. On the contrary, MMseqs can run on shared-memory distributed systems with the help of MPI and OpenMP while PASTIS is fully parallelized and optimized for purely distributed systems. For reference, with the use of sparse matrices, PASTIS can compare 313 million sequences on 2,000 nodes in ∼4 h, sustaining a rate of 320 million alignments per second.

Once an SSN has been created, one can apply a graph-based clustering algorithm to group proteins into families. Despite the great variety of graph-based clustering algorithms available today (Xu and Wunsch, 2005; Brohée and van Helden, 2006; Moschopoulos et al., 2011; Koutrouli et al., 2020b; Karatzas et al., 2021b), only a few can cope with networks of millions of nodes and edges. For example, SPICi (Jiang and Singh, 2010) is a fast, local clustering algorithm that detects densely connected communities within a network. It is one of the fastest graph-based clustering algorithms with *O(VlogV + E)* time and *O(E)* memory asymptotic performance, where *V* and *E* are the number of vertices and edges of the network, respectively. While SPICi has great performance, it is tailored to analyze dense networks. Louvain (Blondel et al., 2008) is a greedy clustering method for identifying communities in large scale networks and while the exact computational complexity of the method is not known, evidence points to *O(VlogV)* time performance. Molecular Complex Detection (MCODE) (Bader and Hogue, 2003) finds densely connected regions in large protein–protein interaction (PPI) networks with polynomial time complexity *O*(*VEd* ^*3*^)*,* where *d* is the vertex size of the average vertex neighborhood in the input graph. Restricted neighborhood search clustering (RNSC) (Biswas and Mukhopadhyay, 2014) uses stochastic local searching and tries to achieve an optimal clustering cost by assigning cost functions to the set of clusters of a graph, requiring *O*(*V* ^*2*^) memory. Affinity-propagation (Frey and Dueck, 2007) is a clustering algorithm based on the concept of “message passing” between data points and is able to cluster 25.000 data points in a few hours, or 120.000 data points in less than a day. The latter achieves performance of *O*(*kV* ^*2*^), where *k* is the number of iterations.

Despite the continuously active research in the field and new methods appearing in the literature, MCL has been one of the most promising algorithms. MCL uses random walks to detect clustered structures in graphs with a mathematical bootstrapping procedure and was initially used to detect protein families and protein interaction modules from sequence similarity information (Pereira-Leal et al., 2004). HipMCL (Azad et al., 2018), is a scalable distributed-memory parallel implementation of the MCL algorithm that, in contrast to previous work, takes advantage of the aggregate memory available in all computing nodes. The unprecedented scalability of HipMCL stems from the use of state-of-the-art parallel algorithms for sparse matrix manipulation. HipMCL is written using the MPI and OpenMP programming interfaces, with the principal aim to speed up graph clustering and efficiently detect clusters on a very large scale. Notably, MCL’s core has remained intact, making HipMCL a state-of-the-art parallel implementation of the original MCL algorithm. For reference, the HipMCL allowed a network clustering of 300 million nodes and ∼17 billion edges in only ∼6 h using ∼136,000 cores.

For higher quality clusters, users are encouraged to filter by alignment length bidirectionally (query vs. target and target vs. query) as well as by applying a similarity or identity threshold during the SSN generation. Notably, homology is inferred based on sequence similarity and homologous sequences usually can have similar functions (Stormo, 2009), whereas more than 90% of all protein pairs with a sequence identity larger than 30% are found to be structurally similar (Rost, 1999). Finally filtering by similarity or identity percentage as well as by alignment length will make the SSN sparser as many of the edges will be discarded. As a result, the SSN’s topology will be further defined in order for the clustering algorithm to detect any densely connected regions. Running a clustering algorithm in an unfiltered SSN would be pointless as it will consider the network as a fully connected graph (clique); thus the higher the similarity threshold, the higher the probability of generating more but more fragmented clusters.

### 4.3 Hierarchical clustering

Hierarchical clustering is a non-graph-based clustering methodology that presents clusters in a hierarchy, often visualized as a dendrogram (Pavlopoulos et al., 2010; Koutrouli et al., 2020b). There are two main strategies to calculate the clusters; *i)* the agglomerative approach, where all sequences start as individual clusters, which are then merged in every iteration step, and *ii)* the divisive approach [DIANA algorithm (Patnaik et al., 2016)], where all sequences start as one cluster and iteratively break into smaller groups. To calculate the various clusters, a full distance matrix without gaps is required. The distance matrix is symmetric, and is calculated as: *1-sequence similarity matrix* and has size *n(n-1)/2* where *n* is the number of sequences.

Widely used agglomerative hierarchical clustering algorithms include the single-, complete-, centroid- and average-linkage methods, as well as neighbor joining (Saitou and Nei, 1987) and the unweighted pair group method with arithmetic mean (UPGMA) algorithms (Day and Edelsbrunner, 1984). The single-linkage algorithm calculates the smallest distance among sequences in each iteration step, whereas the complete-linkage algorithm calculates the longest distance. Centroid linkage algorithms calculate the distance between the centroids of clusters. Average-linkage algorithms use the average distance among all sequence pairs in every iteration step. Neighbor joining (mainly used for the creation of phylogenetic trees) starts with sequences placed in a star-like tree structure and then, at every iteration, a new virtual node representing the two closest sequences is appended as a branch to the tree. UPGMA utilizes the unweighted mean distance between elements of each cluster, meaning that all distances contribute equally to each computed average.

Each iteration of the agglomerative clustering algorithms produces a new level to the output dendrogram (Pavlopoulos et al., 2010). The height at which this dendrogram will be cut is often arbitrarily chosen by the user. However, there are some tools that automate this procedure such as the Dynamic Tree Cut method (Langfelder et al., 2008), which applies a dendrogram cutting threshold according to the shape of the branches. More recently, machine learning techniques such as the PAC Bayesian (McAllester, 1999) have also been applied on dendrogram cutting. Due to the distance matrix necessity and the high running time complexity O (n^3^), hierarchical clustering is not recommended for large-scale analyses.

## 5 Structure prediction

**FIGURE 5:**
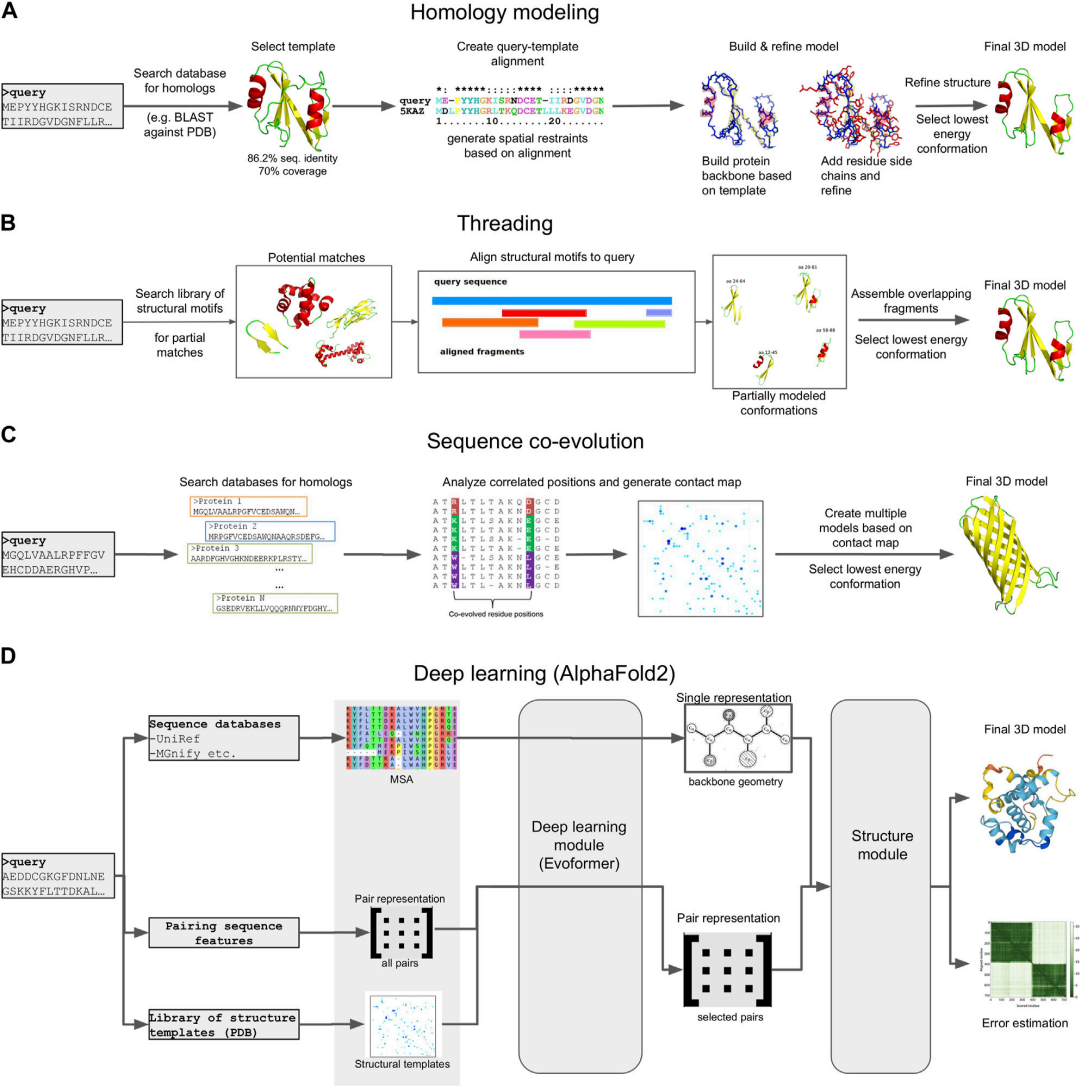
Schematic representation of different 3D modeling approaches. **(A)** Homology modeling. **(B)** Threading. **(C)** Sequence coevolution. **(D)** Deep learning (the AlphaFold2 model is shown as an example).

The function of a protein is directly dependent on its three-dimensional (3D) structure. Through their structures, proteins perform their functions, which range from enzymatic activity and signal transduction to immune responses, DNA replication and transcription and even the mechanical support of the cell (Skolnick et al., 2000). As a result, protein structure determination can be crucial in elucidating the function of metagenome-derived protein sequences, especially in the case of sequences of unknown function, that have no hits to reference genomes or protein family databases. Despite its importance, the experimental determination of protein structures, using techniques such as X-ray crystallography, Nuclear Magnetic Resonance (NMR) or Cryo-electron microscopy, is challenging. In the absence of experimental evidence, computational 3D modeling is a viable means for obtaining mechanistic insight into protein function (Figure 5).

Homology modeling (also known as comparative modeling) is generally the most straightforward approach, provided that template structures with an acceptable sequence identity (>30%) and alignment coverage (>70%) to the target exist (Rost, 1999). The procedure generally involves four steps (Martí-Renom et al., 2000): i) searching the query sequence against a database of templates, typically a subset of the Protein Data Bank (PDB) (Berman et al., 2000) and selecting a target with the best sequence identity and coverage to the query, ii) creating a pairwise sequence alignment between the query and target, iii) mapping the query sequence to the target structure based on the alignment and the satisfaction of spatial restraints (a method based on NMR spectroscopy) and iv), refining the model and selecting the lowest energy conformation. Several computational tools exist for this purpose, with MODELLER (Webb and Sali, 2021), SwissModel (Biasini et al., 2014) and RosettaCM (Song et al., 2013) being the most commonly used.

An alternative to homology modeling, when no adequate homologs exist, is sequence threading, in which prediction is performed by searching the sequence against a library of templates, and “threading” (i.e., placing) each amino acid in the target sequence to a position in each template structure. The template library can contain full-length structural domains, or small fragments extracted from high quality PDB structures, each representing a structural motif (e.g., helix-loop-helix). The best-fit templates are then selected and the query sequence is mapped upon the target structures. Multiple fragments are combined to produce full length configurations, and the lowest energy representation is selected as the final model. Due to this mix and match approach, the derived models generally have a lot of conformation errors, and often require extensive refinement to reach an acceptable state. However, threading has been found to produce models for several targets where no adequate sequence identity with known structures exists, thus complementing homology modeling. Popular tools, either focusing entirely on threading, or offering threading capabilities alongside other modeling methods, include I-TASSER (Yang et al., 2015), Rosetta (Leman et al., 2020), RaptorX (Källberg et al., 2012) and Phyre (Kelley et al., 2015, 2).

Homology modeling and threading are based on two fundamental assumptions: that the number of different folds in nature is fairly small, and that most newly solved structures are likely to have structural domains similar to known folds (Liu et al., 2004). This, however, means that both approaches are unable to predict novel structural folds, i.e., architectures that have not already been determined experimentally. In addition, both methods rely on the target sequence having at least a fraction of sequence similarity (either global or partial) to its structural templates (Baker and Sali, 2001). Despite these limitations, both homology modeling and sequence threading have successfully predicted 3D structural models for metagenomic data. In 2018, Ruppé et al. used homology modeling with MODELLER to produce 3D models for 6,095 antibacterial resistance proteins from the human intestinal microbiome (Ruppé et al., 2019). In 2021, the developers of I-TASSER recruited ∼4.25 billion metagenome sequences from four major biomes to enrich Pfam families, and used threading to predict 3D models for 1,044 domains with unknown structures (Yang P. et al., 2021).

When sequences are not similar to any known template, other *de novo* approaches must be adopted. These include physical interaction-based methods, sequence coevolution analysis and, most recently, deep learning models. Physical interaction-based methods utilize statistical mechanics methods, such as molecular dynamics (MD) or Monte Carlo (MC) simulations (Kroese et al., 2014) to model a protein’s folding path based on its sequence, the physical interactions of the amino acids, and the surrounding environment (e.g., the solvent). Simulating these interactions is based on the use of a “force field,” i.e., a collection of parameters for modeling bonded and non-bonded interactions, usually derived either from high quality experimental measurements or from Quantum Mechanics calculations. A large number of different force fields exist (e.g., CHARMM, AMBER, OPLS, *etc.*) (Robertson et al., 2015; Huang et al., 2017; Tian et al., 2020), and simulations can be performed using high performance tools such as GROMACS (Páll et al., 2020), Desmond (Bowers et al., 2006), NAMD (Phillips et al., 2020) or OpenMM (Eastman et al., 2017), which take advantage of modern hardware capabilities such as parallelization and GPUs. A number of tools that implement specialized MD and MC protocols to guide folding have also been developed, such as QUARK (Xu and Zhang, 2012). Several reports of such simulations successfully reproducing small to medium-sized protein domains, and even a few large proteins, have been reported [reviewed in (Gershenson et al., 2020)]. In addition, MD simulations are the method of choice for Folding@Home (Beberg et al., 2009), one of the largest volunteer-based distributed computing projects for studying protein folding and dynamics. However, while this approach is theoretically very appealing, it can be challenging for large (>150 aa) domains or multi-domain proteins, due to the computational load and the magnitude of the simulation time required to achieve a stable final conformation. What is more, folding simulations perform poorly on categories such as transmembrane proteins, due to the increased complexity of the simulated environment (lipid bilayer). As a result, MD and MC simulations are mostly used in combination with other modeling methods, either to refine and test the stability of the generated 3D models or to explore their structural and functional features under specific conditions (drug binding, effects of mutations, *etc.*).

A complement to physical interaction models is the study of sequence coevolution. The approach is based on the observation that the conserved function of a protein family imposes strong boundaries on sequence variation, and generally ensures a structural similarity for all its members. This means that, in order to maintain energetically favorable interactions, residues in spatial proximity may coevolve across a protein family. Therefore, the correlations of coevolving residues in a sequence alignment of closely related proteins can be used to infer their 3D structure (Altschuh et al., 1987), provided a suitable analysis has been performed. The main input in coevolution modeling is a Multiple Sequence Alignment (MSA), containing proteins belonging to the same family. The alignment positions are scanned using a statistical model to identify correlated positions; notable examples include Direct Coupling Analysis (DCA), mutual information (MI), maximum entropy (ME) and others (Morcos et al., 2011). The inferred positions are then used to generate constraints in the form of a contact map. These constraints are finally used to guide 3D model prediction using existing modeling/threading tools or molecular simulations. Model predictions can be based solely on the restraints of the contact map, or be supplemented by additional analysis of the input sequences, such as secondary structure (Buchan and Jones, 2019) or transmembrane topology predictions (Käll et al., 2007; Hayat et al., 2016). Popular coevolution-based methods primarily include EVfold (Marks et al., 2011) and its successor, EVcouplings (Hopf et al., 2019), a model based on DCA and ME that has been successfully used in multiple case studies, including transmembrane proteins (Hopf et al., 2012; Hayat et al., 2015). Another example is GREMLIN (Ovchinnikov et al., 2014), a pseudo-likelihood maximization (PLM) implementation of DCA that produces constraints compatible with Rosetta. Finally, the C-QUARK pipeline (Mortuza et al., 2021) combines the analysis of ten coevolution algorithms to generate a consensus prediction and guide folding simulations with QUARK.

The popularity of coevolution-based modeling methods has increased during the last decade, mostly due to the increasing number of available protein sequences, which enable generating MSAs suitable for modeling. Especially in the case of metagenomes, the large number of generated sequences has been used to predict the previously unknown structures of several protein families. In 2017, Ovchinnikov et al. successfully predicted the structures of 614 Pfam domains by enriching their profiles with metagenomic sequences from IMG/M and analyzing the enriched MSAs with GREMLIN and Rosetta (Ovchinnikov et al., 2017). Notably, 206 of these models were membrane proteins, while 137 had folds that, at the time, did not exist in the PDB. Similarly, in 2019, the Zhang group used C-QUARK to also model the structures of Pfam domains, with MSAs enriched by metagenomic sequences derived from marine ecosystems (Wang Y. et al., 2019). In both cases, several of the produced models were subsequently validated by experimentally determined structures, demonstrating the validity of the methods.

While the coevolution approach has enabled the modeling of structures that were previously impossible to predict, it is limited by the features of the input alignment. The MSAs must contain an adequate number of members (typically more than 100) with high sequence identity (>90%) and alignment coverage (>75%), in order to successfully infer the required residue correlations. In cases where no such alignments can be provided, the modeling process can fail or produce low quality models. However, a solution to this problem has been recently provided by deep learning-based modeling, i.e., methods utilizing artificial intelligence (A.I.) to *de novo* predict and model 3D structures. This has been made possible thanks to the rise of GPU computing and development of A.I. packages that take advantage of modern hardware capabilities (e.g., TensorFlow) (TensorFlow: Large-scale machine learning on heterogeneous systems, 2015). Like coevolution modeling, the basis of most deep learning methods is an input MSA of proteins belonging to the same family. This can be provided by the user, or automatically created by the method, by searching and retrieving related sequences from databases. At the same time, the MSA’s sequences are searched against a library of structural templates, usually with a sensitive method such as HMMER or MMseqs2, to detect potential remote homologs. The MSA is analyzed to infer correlations between residues positions; however, in contrast to standard coevolution analysis, these correlations are then fed as input to several levels of deep learning modules that iteratively infer structural correlations based on various aspects. This application of A.I. has been found to surpass a lot of the limitations imposed by standard coevolution calculations. The generated restraints are finally used to model a structure, either fully *de novo*, or in combination with restraints from any identified structure templates.

Deep learning models have achieved success at an unprecedented rate compared to all other molecular modeling methods; in fact, the last two Critical Assessment of protein Structure Prediction (CASP) experiments, CASP13 and CASP14, highlighted multiple deep learning models as the most capable *de novo* structure predictors, rivaling experimental approaches (Kryshtafovych et al., 2021). Perhaps the most famous example is DeepMind’s AlphaFold, which, in its current version (AlphaFold2), has achieved a success rate of over 90% in correctly modeling protein structures in CASP14 (Jumper et al., 2021). AlphaFold2 has been used to predict 3D structures for almost the entire human proteome, resulting in more than 20,000 3D models (Tunyasuvunakool et al., 2021). These efforts were later expanded to cover the entire UniProt database. The results of these predictions are hosted in AlphaFoldDB (Varadi et al., 2022), a collaboration between DeepMind and EBI that covers all reference proteomes and currently offers more than 200 million 3D models. The source code of the method has also been made available with an open source license, enabling the development of derivative pipelines. A notable example is ColabFold, which tweaks the original AlphaFold2 workflow to enable running predictions on user-friendly Colab notebooks or local infrastructures rather than large clusters or supercomputers (Mirdita et al., 2022). Other implementations of deep learning methods include RoseTTAFold (Baek et al., 2021) and DeepFold (Pearce et al., 2022). Notably, all of the aforementioned methods utilize metagenomic sequences to build and enrich MSAs during modeling; namely, AlphaFold2 uses MGnify, while RoseTTAFold and DeepFold use MetaClust. More recent developments have also resulted in deep learning methods that predict 3D structures from single sequences, without requiring the generation of an MSA. The premise of these approaches is that since a protein will, typically, fold in a natural setting from its primary amino acid sequence into its three-dimensional structure, MSA analysis should not be required. To achieve this, single-sequence methods are based on deep learning models for natural language processing (NLP) in combination with transformer modules (used by AlphaFold2 and other similar approaches). The two most notable examples are OmegaFold (Wu et al., 2022), developed by Helixon, and ESMfold (Lin et al., 2022), developed by Meta AI Research. Both methods boast comparable performance with AlphaFold2 and RoseTTAFold for their test datasets. In addition, ESMfold was recently used to model 3D structures for more than 600 million metagenome sequences from MGnify, the top 1 million of which are publicly offered through the ESM Metagenomic Atlas database (Lin et al., 2022). However, the soundness of ESMfold and the models hosted in the ESM Atlas have been questioned, both on the accuracy of the method and on the overall quality of the input sequences and produced models (Callaway, 2022).

## 6 Cluster analysis and annotation

### 6.1 Sequence alignments and profiles

The result of clustering is the organization of metagenomic sequences into clusters based on their similarity. These clusters can then be used to create Multiple Sequence Alignments (MSAs), enabling more refined searches against databases, as well as providing the clusters and their components with additional annotation capabilities. MSAs can be created using a combination of various approaches, such as dynamic programming, hierarchical tree building, profile-profile comparisons or Hidden Markov Models (HMMs). MUSCLE (Edgar, 2004) is one of the first alignment tools to implement a profile-profile alignment approach, resulting in high quality MSAs. Clustal Omega (Sievers et al., 2011), the successor of ClustalW/ClustalX, uses seeded guide trees and HMM-based profile-profile alignments to generate alignments for thousands of sequences, and is suitable for medium-length sequences and MSAs. The Kalign algorithm (Lassmann and Sonnhammer, 2005) works by translating protein sequences to a reduced alphabet, using a SIMD (single instruction, multiple data) accelerated, bit-parallel string matching algorithm to compute pairwise distances and applying a Clustal-like approach to construct seeded guide trees. This combination makes Kalign ideal for the fast, parallelizable alignment of distant (low homology) sequences. MAFFT (Katoh and Standley, 2013) uses Fast-Fourier transformations to align thousands of sequences within a few hours, providing both a fast-greedy and an exhaustive mode. PRANK (Löytynoja, 2014) is a phylogeny-aware multiple sequence aligner which makes use of evolutionary information to help place insertions and deletions using the PRANK method. Finally, T-Coffee (Di Tommaso et al., 2011) takes into account structural and homology information to align sequences and offers a number of specialized implementations for specific case studies, such as position-specific iteration (PSI) alignment (PSI-coffee) or transmembrane protein-focused alignment (TM-coffee).

The resulting MSAs of the protein clusters need to be evaluated on their quality in order to be usable. Features that need to be estimated primarily include the MSA’s maximum sequence identity, minimum alignment coverage, pairwise distance distribution and column (position-specific) occupancy (i.e., the percentage of each MSA column covered by sequence residues, not gaps). Additional metrics that are also used in some situations, such as alignment density or Shannon entropy, are derived from the aforementioned features. A good quality MSA is expected to have high column occupancy (and as a result, a high density) throughout its length, and high (>70%) alignment coverage (Valdar, 2002). At the same time, it is expected to have a reasonable maximum sequence identity, high enough to accurately model the evolutionary relationships of the sequences in the cluster (≥ ∼30% typically indicates protein homology) but not so high that it leads to overfitting. This is especially important in cases where an MSA needs to be used as input for analysis [e.g., molecular phylogenetic inference (Kapli et al., 2020) or sequence coevolution (Altschuh et al., 1987)], to train sequence profiles, or to predict 3D structure models (see section “Structure Prediction”).

A reasonable rule, in this regard, is followed by Pfam, whose profiles are represented by full MSAs (containing all sequences in the family) and non-redundant subsets (“seed MSAs”), having a maximum sequence identity of 80%; the latter are also used to construct the families’ HMM profiles (see below). While different aligners might come with slightly different results, one can use alignment correctors to discard underrepresented columns or rows. Characteristic applications for this task are ClipKIT (Steenwyk et al., 2020), BMGE (Criscuolo and Gribaldo, 2010), Gblocks (Talavera and Castresana, 2007), trimAl (Capella-Gutiérrez et al., 2009) and Noisy (Dress et al., 2008). In addition, the HH-suite includes a dedicated tool for MSA filtering and trimming (*hhfilter*) (Steinegger et al., 2019a), capable of producing ready-to-use MSAs for tasks such as phylogeny analysis or 3D structure prediction with deep learning methods such as AlphaFold2.

Refined MSAs can be used as inputs to calculate specialized models, enabling more refined sequence searches that can detect remote homologs. The simplest form of these are sequence motifs, usually formatted as PROSITE patterns (Sigrist et al., 2013) or regular expression sequences. More refined models include position-specific scoring matrices (PSSMs) and HMM profiles. PSSMs can be created using in-house scripts, programming language modules (e.g., Biopython) and even some sequence alignment tools (e.g., T-coffee). The resulting models can be used as input for more sensitive, PSI-based searches in sequence databases with tools such as BLAST (Altschul et al., 1990) (PSI-BLAST), replacing the default substitution matrices (BLOSUM, PAM, *etc.*) to provide search results tailored to the input profile.

Contrary to PSSMs, in which probabilities are computed for each MSA column individually, HMM profiles model MSAs as Markov chains with hidden states, in which the condition of each state is directly dependent on the condition of its previous state. HMM states are annotated with a series of transition and emission probabilities, accounting both for residue occurrences and for the existence of alignment gaps. The latter is especially important as, with HMMs, alignment scoring and gap penalties are tailored to the underlying model itself, rather than being calculated by arbitrary presets (i.e., substitution matrices and pre-defined gap costs). This allows for even more sensitive sequence queries and enables the detection of remotely similar homologous sequences (identity <20%). MSAs, PSSMs and HMMs can also be used to generate the cluster’s consensus sequence, i.e., a representative sequence of the MSA, containing in each position the most commonly found residue in the underlying model. Another useful annotation that can be generated is the cluster’s Sequence Logo, a graphical display of an MSA or HMM consisting of color-coded stacks of letters representing amino acids at successive positions. Sequence Logos provide a richer and more precise description of sequence similarity than consensus sequences and can rapidly reveal significant features of the alignment that could otherwise be difficult to perceive. Popular tools that can generate Sequence Logos from MSAs or profiles include WebLogo (Crooks et al., 2004), HMMLogo (Eddy, 2011) and Skylign (Wheeler et al., 2014).

The two most popular packages for the creation and use of HMM profiles are HMMER (Finn et al., 2011) and HH-suite (Steinegger et al., 2019a). The HMMER package provides tools for the training of HMM profiles from input MSAs (*hmmbuild*), profile-based multiple sequence alignment (*hmmalign*), sequence-sequence (*phmmer*, *jackhmmer*) and sequence-HMM searches and tools to generate annotations, including sequence logos (*hmmlogo*) and consensus sequences (*hmmemit*). Notably, HMMER is the standard tool used by most of the currently prominent protein family databases (Pfam, InterPro, *etc.*), which adopt the package’s file format as the native format of their models. Similar to HMMER, the HH-suite provides tools for creating HMMs (*hhmake*) and performing queries against reference databases (*hhblits*, *hhsearch*), albeit in a different format than HMMER. However, in addition to sequence-HMM queries, HH-suite also allows performing profile-profile alignments, enabling even more sensitive sequence searches. The generated HMM profiles from both tools can be used to search and detect remote homologs in reference databases, including both sequence databases such as UniProt or RefSeq and specialized protein family collections such as Pfam (Mistry et al., 2021), COG (Galperin et al., 2021), or InterPro (Blum et al., 2021). Notably, InterPro provides its own dedicated search tool [InterProScan (Jones et al., 2014)] for searching its database components, which now include major protein family databases such as Pfam, TIGRFAMS (Haft et al., 2003), CATH-Gene3D (Sillitoe et al., 2021) and PROSITE (Sigrist et al., 2013). Database hits detected through profile-based searches can be used to functionally annotate the source sequence clusters; this enables the functional characterization of clusters formed by unknown sequences that had no hits during the gene calling and annotation step. In addition, the derived HMMs can be further used to search metagenomic sequence datasets and recruit additional sequences for the underlying clusters; this can help increase cluster size, and provide additional annotation to the ever-increasing metagenome sequence space.

### 6.2 Structure searches and functional annotation

**FIGURE 6:**
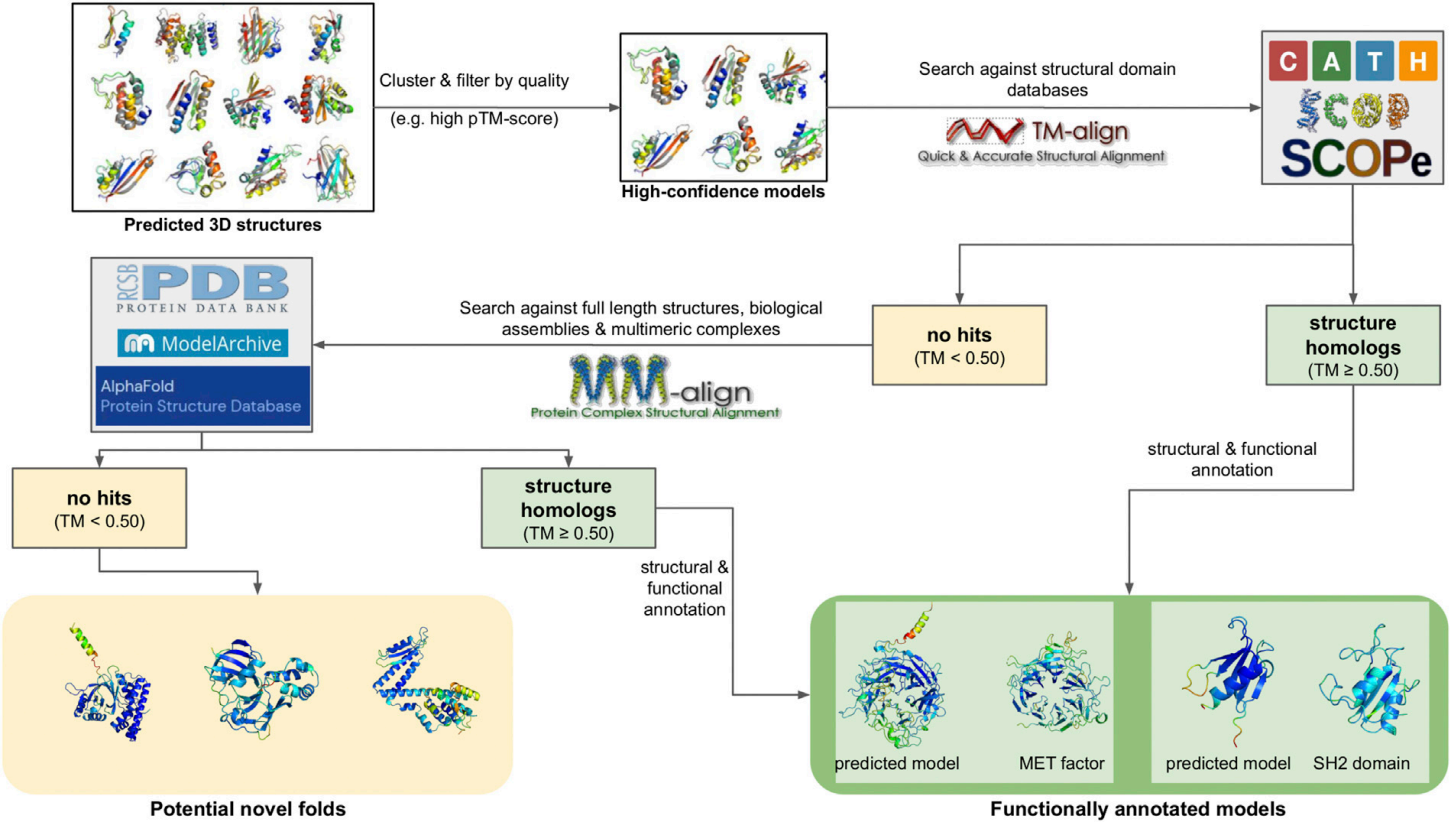
Example structure search and functional annotation for a set of predicted 3D structures. In the first step, the models are filtered to keep only high-quality models, typically represented by a high predicted TM-score (pTM) value. The models are also clustered based on their structural similarity. The high quality, non-redundant set of models can then be searched against databases of structural domains (e.g., CATH-Gene3D, SCOP and SCOPe) with a fast, TM-score based method such as TM-align. Models with significant hits (TM-score ≥0.50) are functionally annotated based on their structural homologs. Models with no hits (TM-score <0.50) are further searched against databases of full-length structures (containing one or multiple domains), biological assemblies or protein-protein complexes (PDB, ModelArchive, AlphaFoldDB, *etc.*) with a multimeric complex-enabled search method such as MM-align. Again, models with significant hits are functionally annotated based on their homologs. Finally, models with no hits to any structural database can be considered as potential novel folds.

The produced MSAs of the clusters can also be used as input for the generation of 3D structure models. Various types of approaches may be followed, described in detail in Section 5 (“Structure Prediction”) of this review. Regardless of the method used, the generated 3D structure models can then be searched against repositories of 3D structures to identify potential matches. This can be used to further annotate the functional role of the clusters, particularly in cases with no strong sequence similarity hits, since it is generally accepted that protein structure is more conserved than protein sequence, and that the structure of a protein essentially defines its function (Figure 6). The most prominent reference database to be searched is the Protein Data Bank (PDB) (Berman et al., 2000), the collection of all experimentally determined protein structures. In its current version (February 2023 data), the PDB contains ∼202,000 deposited 3D structures. In addition, the database contains over 200,000 biological assemblies, i.e., multimeric configurations based on the crystal symmetry of the aforementioned data. In addition to the PDB, searches can be performed against publicly available databases containing theoretical 3D models. The most prominent examples of such databases include AlphaFoldDB (Varadi et al., 2022), which contains structure predictions performed by AlphaFold2 and the ModelArchive (Schwede et al., 2009), a collection of predicted 3D structure models from publications. It should be noted that the structural data in these databases is redundant, meaning that a single protein may be represented by multiple structures, determined for multiple organisms, at varying levels of resolution, in different conformational states or in complexes with different interacting partners or chemical compounds. In addition, as the number of unique protein structural folds in nature is fairly small, most structural domains are present in a large number of structures, and represented by multiple entries in the databases. For this reason, it is faster and often more useful to perform searches against non-redundant sets, either subsets of the PDB clusters at various levels of sequence identity or structure family databases like CATH-Gene3D (Sillitoe et al., 2021), SCOP (Andreeva et al., 2020) and SCOP Extended (SCOPe) (Chandonia et al., 2022). The latter are non-redundant collections of structural domains, clustered based on structural architecture, with each CATH-Gene3D or SCOP/SCOPe family represented by a single, high quality domain structure.

Structure-based searches are usually performed by structure alignment or superposition, i.e., fitting the query structure against its target in 3D space and evaluating the similarity of the two. Similarity can be measured using two different criteria, the Root Mean Square Deviation (RMSD) or the Template Modeling score (TM-score). RMSD is the measure of the average distance between the atoms (usually the backbone) of two superimposed proteins, with higher RMSD values (typically measured in Å or nm) indicating greater diversity. RMSD-based queries can be performed using a large number of protein structure alignment tools, notable examples of which are the Dali (Holm, 2022) and FATCAT (Li et al., 2020) web servers. Dali works by splitting the input query and target structures into hexapeptide fragments and then calculating a distance matrix for each structure, through the understanding of the contact pattern between successive fragments. If two proteins’ distance matrices are the same or share similar features in almost the same positions, they can be said to have similar folds and length loops connecting the secondary structure elements. FATCAT works by representing each structure as a contact map and then comparing the two maps for the existence of statistically significant similarities or differences. In addition, the algorithm takes into consideration potential flexible protein segments (e.g., hinges) that could result in conformational transitions for otherwise similar proteins and produce high RMSD values if the structures were considered as completely rigid.

However, because RMSD is computed with equal weight over all residue pairs, a large local error on a few residue pairs can result in quite large deviations, even when the global topologies of the compared structures are actually similar. In addition, it is highly dependent on protein length, meaning that RMSD comparisons between proteins with significant length differences are essentially meaningless. Finally, a lot of RMSD-based tools rely on preliminary sequence alignments to guide structure superposition, meaning that they cannot be used in cases where the query has no significant sequence homologs. An alternative to RMSD is the TM-score, defined as a variation of the Levitt-Gerstein (LG) score, which weights shorter distances between corresponding residues more strongly than longer distances (Zhang and Skolnick, 2004). Therefore, it is more sensitive to the global topology rather than local structural variations. In addition, its value is normalized so that the score magnitude relative to random structures is not dependent on the protein’s size. TM-score values range from 0.0 to 1.0, with scores <0.2 corresponding to randomly chosen unrelated proteins, whereas TM-score values >0.5 indicate proteins belonging to the same structural family. The most prominent TM-score based alignment method is TM-align (Zhang and Skolnick, 2005), which relies on dynamic programming to align the secondary structures of the query and target and does not depend upon sequence similarity, meaning that it can be used to compare distantly related proteins. TM-align is very fast and can be integrated into user-made scripts or pipelines, so that it can be executed in parallel to concurrently perform multiple pairwise queries. Variants of TM-align have also been developed, including MM-align (Mukherjee and Zhang, 2009), a variation capable of performing alignments featuring multimeric complexes as well as single structures, and mTM-align (Dong et al., 2018), which can perform massive structure queries against reference databases, as well as multiple structure alignments.

Finally, one recently developed structure search method that is quickly gaining ground is FoldSeek (Kempen et al., 2022). Contrary to the aforementioned tools, FoldSeek does not work through standard structure superposition or RMSD and TM-score, although it can compute both scores for consistency with other methods. Instead, the FoldSeek approach works by representing protein tertiary interactions as sequences over a structural alphabet and comparing structures using sequence alignments with the double-diagonal *k*-mer-based prefilter and gapless alignment prefilter modules from MMseqs2 (Kempen et al., 2022).

### 6.3 Gene neighborhood inference

The vast majority of cellular functions are not conducted by one protein alone but by multiple proteins, co-operating in various manners. In the majority of genomes that have been studied, the positions of the co-regulated genes encoding these proteins are not random; instead, genes participating in the same process are almost always co-localized and organized in various types of clusters, collectively known as gene neighborhoods. This phenomenon is especially prevalent in bacteria and archaea (Santangelo et al., 2008), as well as some fungi such as yeast (Poyatos and Hurst, 2007), where genes participating in the same function are organized in clusters known as operons and are co-transcribed and co-translated (Jacob, 2011). However, organized gene clusters (both operon-like and other forms) have also been observed in the genomes of multicellular organisms (Lee and Sonnhammer, 2003), including the human (Mégy et al., 2003), other mammalian (Fukuoka et al., 2004; Carninci et al., 2005), insect (Boutanaev et al., 2002), worm (Blumenthal et al., 2002) and plant genomes (Tang et al., 2008). Analyzing gene neighborhoods can help detect genes participating in common processes, predict protein-protein interactions and, in the case of novel, uncharacterized (“orphan”) genes, infer their potential function by evaluating the functions of their neighboring genes (Huynen et al., 2000). Gene neighborhood analysis and the study of gene synteny is commonly used in studying genomic structure (Wolf et al., 2001). More recently, gene neighborhood analysis has been applied to the study of metagenomes, enabling the construction and visualization of functional gene networks (Aßhauer et al., 2014; Kim and Lee, 2017; Brown et al., 2020).

**FIGURE 7:**
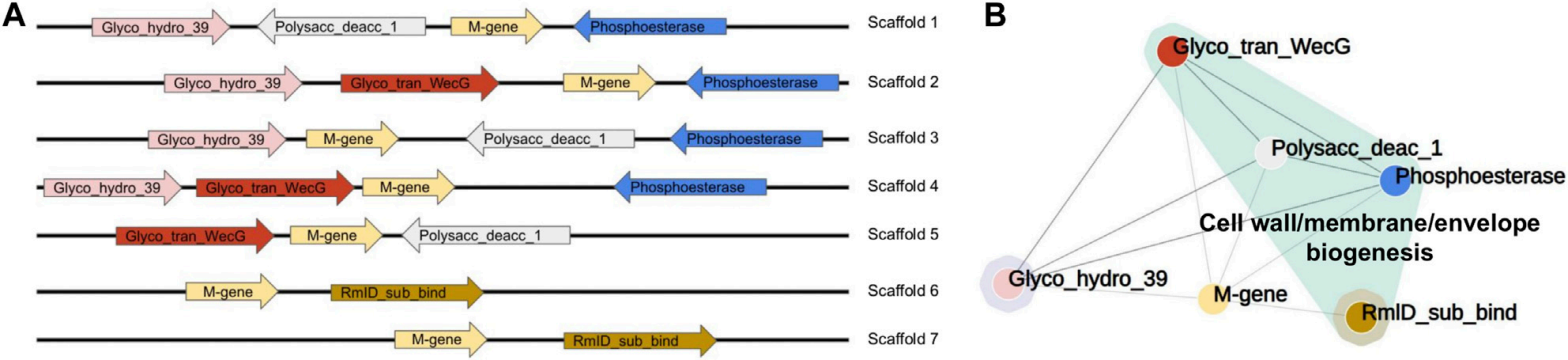
Example of a gene neighborhood analysis for a cluster of unannotated metagenome sequences, represented as “M-gene”. **(A)** Simplified visualization of a synteny analysis for seven metagenome scaffolds, containing members of the M-gene cluster. Each gene is represented by an arrow and colored differently. The direction of the arrows represents the directionality of the ORFs in each scaffold. In the analyzed scaffolds, the M-gene ORF co-occurs with a number of other protein-coding genes, each corresponding to a Pfam domain. **(B)** Gene co-occurrence network, based on the results of the synteny analysis. Each node represents a protein-coding gene and is colored using the same scheme as in **(A)**. Edges (interactions) between nodes are derived based on the co-occurrence of their genes in the same scaffold. As it can be seen, the unannotated metagenomic cluster (M-gene) co-occurs with a tightly connected group of Pfam domains (Phosphoesterase, Glyco_tran_WecG, Polysacc_deacc_1 and Glyco_hydro_39), which are all found in the same scaffolds alongside M-gene members. In addition, M-gene co-occurs with RmID_sub_bind. Notably, four of the co-occurring protein domains are in the same functional category (Cell wall/membrane/envelope biogenesis), as indicated by their annotation in COG. This could mean that the unannotated M-gene cluster may participate in this function as well. The network was constructed using NORMA (Karatzas et al., 2022b).

In its simplest form, identifying the neighbors of a metagenome cluster representing a protein family can be performed simply by identifying the neighboring genes of the cluster’s members, based on their positions in the source metagenomic contigs and their distance from the coordinates of the genes forming the cluster (Figure 7). By compiling these neighbors, mapping them to reference databases such as Pfam or COG and inferring their function, the gene neighborhood of the cluster can be constructed, and provide hints towards the cluster’s functional role. This can be especially useful for the clusters of uncharacterized sequences, with no hits to any reference database or known protein family. In addition, gene neighborhood analysis can be used to predict biomolecular interactions involving the proteins represented by the cluster in various contexts (protein-protein, protein-chemical, host-pathogen, gene-disease interactions, *etc.*), by linking the produced gene neighborhood with annotation from various biomolecular interaction databases [reviewed in (Baltoumas et al., 2021a)]. Simple distance based calculations can be performed using the coding sequence coordinates of the contigs, produced during the gene calling stage of a metagenomic analysis. More detailed inference can also be performed using specialized tools designed to analyze genomic structure and gene position patterns; examples include general purpose tools such as G-NEST (Lemay et al., 2012) and the JAX Synteny browser (Kolishovski et al., 2019), as well as metagenome-focused implementations such as FeGenie (Garber et al., 2020) and the EFI enzymology tools (Zallot et al., 2019).

### 6.4 Ecosystem annotation and distribution

Previous sections in this review have mostly focused on analyzing and annotating the sequence, emphasizing structural and functional aspects of metagenomic sequences and their clusters. However, ecosystem annotation is equally important, as a key feature of metagenomics is the study of biodiversity, understood partly by examining the environmental properties of the analyzed samples. In the context of protein family biodiversity exploration, the protein space can be divided according to metagenomic sample source environments. This can be beneficial in a reciprocal fashion: *i),* protein families can be profiled according to the environment from which their member proteins originate, and *ii),* different types of environments can be characterized according to their protein family richness.

Prior to any computation, metagenomic sequences inherit the contextual information of the sample from which they originated. A sample’s isolation source, for example, describes the environment from which a sample was collected. Spatial, temporal and other characteristics of the sampling environment are key both in interpreting unknown genes and in obtaining new insights about known ones (Nayfach et al., 2021). The experimental procedures through which metagenomic sequences have been obtained are also key pieces of background information. The richer and more comprehensive such contextual pieces of information are, the stronger the link among a study and its sequences becomes. Such a link can be used from a single-study *search and retrieve* operation, to integrative queries and multiple-study comparative analyses. However, in order for this annotation to be useful, it needs to be formatted in a standardized, accessible and easy to use format, preferably in line with established FAIR (Findability, Accessibility, Interoperability, and Reusability) principles (Wilkinson et al., 2016).

For standardization to move from wishful thinking into reality, accurate, well-structured and semantically concise metadata are key for describing a metagenomic sample’s context. Environment Ontology terms, for example, can describe a sample’s environment both in a broad context (*biome*), its *material*, and more fine-grained characteristics (*feature*). Taxonomy data structures like the NCBI Taxonomy (Schoch et al., 2020; Sayers et al., 2022) can describe host information in *host-associated* metagenomic samples. In this case, anatomy ontologies like Uberon (Mungall et al., 2012) and Brenda Tissue Ontology (Gremse et al., 2011) can add collecting tissue descriptors. Disease modeling knowledge structures like the Disease Ontology (Schriml et al., 2012) can capture the health or disease host status. Initiatives such as the National Microbiome Data Collaborative (NMDC) (Yilmaz et al., 2011; Mirzayi et al., 2021; Vangay et al., 2021) are promoting the uptake of standardized contextual metadata by the community *via* detailed example-containing checklists and best practice guidelines. Ontology annotation suggestion tools, such as BioSamples/ZOOMA (Courtot et al., 2022) and EXTRACT (Pafilis et al., 2016), can also assist metadata enrichment. However, despite the existence, irrespective of any shortcomings, of the related knowledge structures, software, and community actions, incomplete or inaccurate sample metadata remain (Nassar et al., 2022)**.**

In metagenomics, the most used biome classification systems are the GOLD database’s ecosystem classification (Ivanova et al., 2010), the Environment Ontology (ENVO) (Buttigieg et al., 2016) and the Earth Microbiome Project Ontology (EMPO) (Shaffer et al., 2022). GOLD uses a five-level hierarchical system to organize metagenomes based on their source biome (*Ecosystem- > Ecosystem Category- > Ecosystem Type- > Ecosystem Subtype- > Specific Ecosystem*). At the top level, metagenomic datasets are grouped into three main ecosystems (“*Environmental*”, “*Host-associated*” and “*Engineered*”), each of which is then further divided into subcategories based on biome aspects, as well as taking into account knowledge of key variables that influence community composition. These have been defined using a mixture of sources; specifically, the Environmental and Host-associated top-level groups are based on the equivalent categories used by GenBank (*Ecological* and *Organismal*). Environmental communities are separated by the ecosystem category (*aquatic*, *terrestrial*, *air*) and ecosystem type (e.g., *freshwater*, *marine*) with more detailed categorizations based on specific features (e.g., *salinity*, *pH*). Host-associated datasets are defined by host phylogeny, based on the NCBI taxonomy system, then sampling site (e.g., *digestive system*, *respiratory system*). Finally, GOLD includes a distinct category (“*Engineered*”) that separates manipulated communities such as *bioreactors* or *treatment plants*; this helps highlight the differences in metagenomic communities that occur in these systems, compared to natural environmental communities (Mukherjee et al., 2022).

The Environment Ontology (ENVO) is a community-led ontology that represents environmental entities, features and materials (Buttigieg et al., 2016). In its initial form, it started as a relatively simple, controlled and structured vocabulary to support the metadata checklists of the Genomics Standard Consortium (GSC). However, it has matured into a fully-fledged, FAIR-compliant ontology, offering representations of biomes, environmental processes and entities relevant to environmental health initiatives. Similar to other ontologies (e.g., GO), terms in ENVO represent a controlled vocabulary and are organized in a hierarchical manner. ENVO’s terms can describe a sample’s environment both in a broad context (*biome*), its *material*, and more fine-grained characteristics (*feature*). For this reason, ENVO has become a recommended standard for the minimum information on genomic, metagenomic and marker gene sequences (MIGS, MIMS and MIMARKS) (Kottmann et al., 2008), as per the instructions of the Genomics Standards Consortium (GSC). ENVO broad scale terms are used to describe biomes (e.g., *forest biome*, *oceanic biome*, *etc.*), local scales are used to describe features (e.g., *mountain*, *river*), and mediums are used to describe materials (e.g., *soil*, *water*) when annotating the biome of a submitted metagenome.

The Earth Microbiome Project (EMP) is a collaborative effort aimed at sampling Earth’s microbial communities at a large scale, to construct a global gene atlas describing protein space, environmental metabolic models for each biome, and a global metabolic model (Thompson et al., 2017). The project has delivered an analysis of approximately 500,000 reconstructed microbial genomes and has provided the scientific community with a number of metagenome sampling, processing and analysis protocols, including a dedicated ontology (EMPO) for the biome characterization of metagenomic samples. EMPO is organized into four levels (Shaffer et al., 2022), the first three of which describe a sample on the basis of host association (*Free-living* or *Host-associated*), salinity (*Saline* or *Non-saline*), and host taxon/phase (*Solid*, *Aqueous*, *Plant*, *Animal, etc.*), while the fourth, recently added, annotates the precise source type of the dataset (*e.g., Animal Gut*). EMPO is a continuously evolving project, expected to grow and expand as metagenomic datasets from more diverse biomes become available.

A comparison of the GOLD, ENVO and EMPO classification systems reveals that all three alternatives have their strengths and weaknesses. GOLD is currently the most diverse and inclusive biome classification system to date, and remains unique in integrating environmental, host-associated, and engineered habitats in a single ontology (Mukherjee et al., 2022). As a result, both IMG/M and MGnify use GOLD as the main biome classification system for their datasets. However, compared to ENVO, GOLD lacks several of the standardized features of FAIR-compliant ontologies and is not as adaptable. ENVO has a more structured organization that can be easily adapted and expanded as needed; this is evidenced by the already enormous evolution of the ontology (Buttigieg et al., 2016). For this reason, ENVO terms are regularly used in the description of environments in MGnify and MG-RAST, and efforts have been made to map GOLD ecosystems to ENVO terms. A possible complexity when working with ENVO is that it interoperates with other ontologies. For example, withhost-associated samples, the consideration of NCBI Taxonomy Identifiers in study MIxS host fields, or of anatomy ontology terms (like ÜBERON ones) for the anatomical part of the host might be needed. Finally, the EMPO ontology appears as a compromise of the two; it adopts a classification scheme somewhat similar to GOLD (although it lacks a distinct group for *Engineered* biomes) and, at the same time, a strict ontology format; in addition, EMPO terms have been mapped to their ENVO counterparts since the very first implementation of the ontology. However, it remains limited, having no deeper classification levels that could enable annotating a sample to the level of detail offered by other ontologies.

In addition to environmental classification information, available metadata can be retrieved upon sequence download from the data repositories like MGnify (Mitchell et al., 2019), SRA (Kodama et al., 2012), IMG/M (Chen et al., 2022), IMG/VR (Camargo et al., 2022) and MG-RAST (Meyer et al., 2019). Literature-extracted metagenomic study metadata, for studies available in MGnify and linked-literature in EuroPMC, (Nassar et al., 2022), can also be retrieved. *Ad hoc* mining of metagenomic study literature and free-text metadata fields is also possible with tools like EXTRACT (Pafilis et al., 2016), OnTheFly2.0 (Baltoumas et al., 2021b)**,** Darling (Karatzas et al., 2022a), and BioSamples/ZOOMA (for metadata fields) (Courtot et al., 2022). Finally, the PREGO (Process, Environment, Organism) resource (Zafeiropoulos et al., 2022) can be used to showcase, analyze, and combine extracted pieces of environmental information to address integrative molecular ecology questions.

### 6.5 Strategies for organizing families into possible superfamilies

Following their annotation, protein family clusters can be further grouped into larger superclusters or superfamilies. A protein superfamily (also known as a *clan*, although the term is usually applied to enzymes) is the largest grouping of proteins for which common ancestry can be inferred. Superfamilies typically contain several protein families that show sequence similarity within each family. These families can be grouped together in the same group by a number of features, such as: *i)* distant sequence similarities (sequence-based), *ii)* phylogenetic relations, *iii)* structural homology (structure-based) or *iv)* common function.

In its simplest form, sequence-based superfamily grouping is performed using pairwise sequence or profile alignments. The first can be done by using the families’ consensus sequences and performing an all-against-all comparison. Alternatively, one can perform the same task using profile-profile alignments, either at the MSA level with MUSCLE (Edgar, 2004), or at the HMM level with HH-suite (Steinegger et al., 2019a).

Simple sequence-based organization can be further enhanced by exploring the evolutionary relations of the proteins through phylogeny inference. By phylogenetic analysis, protein clusters can be further organized into clades, reaching back to their most distant common ancestor. Such an analysis can be performed using statistical methods, such as Bayesian inference, both with standard tools like MrBayes (Ronquist et al., 2012) and with metagenome-focused pipelines such as BiomeNet (Shafiei et al., 2014).

Another more robust way to create superfamilies is to use structure-based clustering, since protein structures are generally more conserved than sequences and, therefore, sequences with low sequence identity may actually adopt the same fold. By performing structural alignments, and grouping structures based on their similarity rather than sequence identity, structures representing protein families can be grouped into higher order categories. This is the basis for the organization of protein structures in families and superfamilies in structural domain databases such as CATH-Gene3D (Sillitoe et al., 2021) or SCOP (Lo Conte, 2000; Andreeva et al., 2020); in addition, a number of metagenome-enriched 3D structure modeling projects have applied the same methodology (Ovchinnikov et al., 2017; Wang Y. et al., 2019). In the concept of metagenome clusters, superfamily organization can be performed by performing all vs. all structural alignments, either manually or with tools such as TMalign or MMalign, and selecting a metric capable of distinguishing structural homology, such as the TM-score (typically, protein structures with TM-score >0.50 are considered part of the same structural family).

In contrast to the above options, which rely exclusively on protein sequence/structure features, functional clustering refers to grouping proteins in families or superfamilies based on their functional annotation. This process is, to some extent, related to structure-based clustering, as proteins sharing the same fold likely perform similar functions. However, this is not always the case, as some superfamilies may include functionally relevant but structurally more diverse members. Functional clustering can be performed by matching cluster members to functional terms, usually in the form of controlled vocabularies, such as Gene Ontology (GO) terms (The Gene Ontology Consortium et al., 2021), KEGG Orthology (KO) pathways (Kanehisa and Sato, 2020), or COG functional categories (Galperin et al., 2021). This matching is typically performed during the gene calling stage of a metagenomic analysis; the clustered sequences can then be analyzed to identify the most overrepresented functional terms of their group, usually with statistical analyses offered by functional enrichment tools (Subramanian et al., 2005; Schölz et al., 2015; Liao et al., 2019; Thanati et al., 2021).

## 7 Visualization of metagenomic data at a raw and family level

Data visualization is one of the most crucial and challenging aspects of metagenomic research. Visualization tools can provide a valuable complement to automated workflows and pipelines, enabling researchers to derive scientific insight from large-scale data sets. At the same time, effective visualization can be used to compare datasets from different sources, derive associations between components (e.g., metabolic pathways, signaling mechanisms, *etc.*) and be used as the basis to conduct further, more advanced analyses. In this sense, visualization is not only concerned with the graphical representation of the data, it is also an essential tool for exploratory analysis (Sudarikov et al., 2017).

The choice of using a visualization scheme to display and analyze metagenomic data heavily depends upon both the number of the datasets to inspect and the type of visualization/analysis that needs to be performed. Graphs such as pie charts, bar plots, circos plots, Sankey diagrams or bubble charts can be used to explore taxonomic abundances in metagenomic datasets and compare features between multiple metagenomes, although their visualization capabilities decrease as the number of datasets increases. Similarly, venn diagrams can help plot the relationships (unions, intersections, *etc.*) among a small number of datasets (typically up to five or 6); for larger numbers of datasets, UpSet plots can be a useful alternative. Rarefaction curves can help plot the richness (diversity) of a microbial community, or simulate the growth rate for features such as gene/protein sequences or clusters, based on a background reference. Tree diagrams and dendroscopes can plot taxonomic ranks, phylogeny distribution and even sequence clustering results (e.g., hierarchical clustering) (Sudarikov et al., 2017). Finally, various types of interaction networks can be used to plot and analyze features such as gene co-occurrence, protein-protein interactions, sequence/cluster/dataset-biome relationships, disease annotations and even taxonomic distributions (Koutrouli et al., 2020b).

There are many solutions to generating visualizations such as the ones referenced above. For instance, data plotting can be performed using specialized visualization packages in programming languages such as Python or R. These can be general purpose, such as Plotly (Sievert, 2020) or Matplotlib (Hunter, 2007), designed with biological data in mind, such as the large number of tools offered by Bioconductor (Gentleman et al., 2004) and Biopython (Cock et al., 2009), or even geared towards metagenomes. Examples of the latter include gbtools, an R package that implements methods to visualize metagenome bins by plotting coverage (sequencing depth) and GC values of contigs, and also to annotate the plots with taxonomic information (Seah and Gruber-Vodicka, 2015). A similar tool is QIIME2, a fully functional Python package enabling researchers to start an analysis with raw DNA sequence data and finish with publication-quality figures and statistical results (Bolyen et al., 2019). In contrast to the above tools, which require the user to have at least elementary programming skills, a number of ready-to-use solutions also exist, offering visualization capabilities coupled with user-friendly interfaces. For example, VICTOR is a pipeline enabling the comparison of multiple sets (gene sets, clustering results, *etc.*) with an abundance of visualization options (e.g., bar charts, heat maps, Sankey plots, interaction networks) and statistical metrics (mutual information, adjusted rand index, *etc.*) (Karatzas et al., 2021b). Krona is a frequently used, web-based interactive metagenome visualization platform. It allows the intuitive exploration of relative abundances and confidences within the complex hierarchies of metagenomic classifications. Its rich and interactive displays facilitate more informed interpretations of metagenomic analyses, while its implementation as a browser-based application makes it extremely portable and easily adopted into existing analysis packages (Ondov et al., 2011). Another example is the Workflow Hub for Automated Metagenomic Exploration (WHAM!), an interactive tool capable of user-directed visualization and analysis for multidimensional, shotgun-sequenced metagenome and metatranscriptome datasets (Devlin et al., 2018). MetaG provides a pipeline for analyzing reads from both targeted and whole genome sequencing, coupled with visualization using intuitive, interactive graphs (Chowdhury et al., 2016). MetaViz, an R and NodeJs-based platform, provides a novel navigation tool for exploring hierarchical feature data that is coupled with multiple data visualizations including heatmaps, stacked bar charts, and scatter plots. It also supports a flexible plugin framework, enabling users to develop and add their own visualization tools (Vázquez-Ingelmo et al., 2022). Finally, MetaSee is a Java-based platform, offering the interactive visualization of metagenomic samples of interest at multiple levels (global view, phylogenetic view, sample view and taxa view), and an Application Programming Interface for the development of new analysis and visualization plugins (Song et al., 2012).

In addition to the plotting tools referenced above, several approaches for the visualization and analysis of metagenomes involve the use of interaction networks (e.g., host-microbiome). Multiple implementations for network visualization have been developed and extensively reviewed in the literature (Pavlopoulos et al., 2008; 2011; 2017; 2018; O’Donoghue et al., 2010; Saito et al., 2012; Koutrouli et al., 2020b; 2020a; 2021; Baltoumas et al., 2021a; Karatzas et al., 2022b). In the scope of metagenomics, interaction networks can be used to visualize the relationships between metagenomic components in the form of gene neighborhood networks, metabolic paths and gene-disease associations. Heterogeneous information with metadata from various sources can also be visualized at a network level with the help of multilayered graphs (Karatzas et al., 2021a; Kokoli et al., 2022; Zhou et al., 2022).

## 8 Limitations and challenges

The metagenome world offers a great space for discovering novelty; however, despite the progress that has been made in metagenomics-based investigations, the currently available metagenomic analysis workflows suffer from a number of issues. One crucial and potentially limiting factor is the choice of sequencing technology which, essentially, defines the type of the analysis and influences the quality and content of the results. Amplicon sequencing approaches, such as 16s/18s/ITS rRNA sequencing are established, low cost and low error solutions that can efficiently screen for variants and target organisms, and describe and compare the diversity of multiple complex environments. Such technologies are routinely used in population and microbial community studies and can help study the phylogenetic profiles of the studied microbiomes. However, taxonomic assignment through rRNA sequencing is inherently biased, as it heavily depends on the selected primers and targeted variable regions. Furthermore, the analysis is limited to bacteria and archaea (16s) or fungi (18s and ITS), and only offers a broad taxonomic profile for the samples, reaching, at best, the level of genus. Finally, as these methods focus exclusively on marker RNA regions, they cannot provide any functional profiles for the analyzed microbiomes, except in the form of predicted general functionality, achieved through the use of prediction tools such as PICRUSt (Langille et al., 2013). On the other hand, shotgun metagenomic sequencing, especially its high throughput implementations, encompasses the sequencing of the entire sample content, and offers the capability of advanced taxonomic assignment (provided adequate marker regions or characteristic genes are available), often to the level of species or strain for all domains of life (bacteria, archaea, eukarya and viruses). What is more, shotgun sequencing results can be assembled to MAGs and used for gene calling and advanced functional annotation of the underlying microbial communities, utilizing the wide array of methods described in this review. However, the methodology is prone to errors, resulting in problems such as metagenome fragmentation or host DNA contamination. These, in turn, can produce artifacts in subsequent analysis steps, including taxonomic assignment, gene calling and functional annotation. Despite their drawbacks, both approaches have their merits, and their application heavily depends upon the scope of each metagenomic study (Rausch et al., 2019; Durazzi et al., 2021).

Another critical limitation is the dependence of gene calling on taxonomy for properly choosing the correct translation table and gene structure model. In cases where taxonomic assignment cannot be performed (e.g., because the contigs do not contain any rRNA genes), it is up to the capabilities of the chosen prediction tool to correctly identify the ORFs. This can lead to translation errors and misidentified ORFs, especially in cases of alternative-coded genomes and metagenomes (Dimonaco et al., 2022). Dealing with this limitation involves applying additional filters and prediction tools. For example, IMG/VR re-analyzes metagenomic data with VirFinder (Ren et al., 2017) and custom markers from the Earth Virome workflow (Paez-Espino et al., 2017b) to identify viral contigs (Paez-Espino et al., 2017a; Roux et al., 2021). In addition, a variation of Prodigal, called Prodigal-gv has been recently developed, meant to improve gene calling for giant viruses and viruses that use alternative genetic codes. However, these practices are mostly limited to specific cases and have not yet been adopted by generalized workflows. A related challenge is that currently used gene calling methods are primarily designed for prokaryotic genomes and metagenomes. This means that the quality of their predictions is significantly decreased on eukaryotic sequences, which often contain introns and, generally, have a vastly more complex structure. It should be noted that some eukaryotic-focused gene prediction methods exist, such as AUGUSTUS (Hoff and Stanke, 2019) or GeneMark-ES/ET (Lomsadze et al., 2014), but their performance has mostly been evaluated with regards to complete genomes, not metagenomes. While some metagenome-specific eukaryotic gene predictors have also recently appeared in the literature, such as MetaEuk (Levy Karin et al., 2020) and EukMetaSanity (Neely et al., 2021), they are mostly based on homology searches against reference databases or RNAseq evidence, rather than actually modeling the eukaryotic gene structure. As such, any predicted genes of eukaryotic metagenomes that are not supported by transcriptomic or metatranscriptomic data should be handled with caution.

An additional issue that needs to be considered is the prediction of false gene length, leading to truncated sequences. A significant portion of these incomplete sequences can be detected by the lack of start or stop codons, though using only genes with valid start and stop codons is not going to eliminate the majority of potential gene fragments. Finding the correct start site is a challenging task even when annotating complete genomes, and when dealing with short, error-prone contigs, gene predictors may pick incorrect start sites, oftentimes downstream from the correct start codon. Therefore, unless validation is provided through functional annotation, any gene that does not have another ORF between its start/stop position and the edges of the contig is suspect, and may actually be truncated. Related to the above is the observation that, due to the fragmented nature of metagenomes, protein sequences may be clustered at the very beginning and end of some scaffolds. While this may seem like an artifact, clusters above a certain number of members (e.g., 50 or more) reduce the probability of such a phenomenon to occur by chance. As these sequences are located very close to the contig ends, they may actually be truncated. However, a lot of these “suspect” proteins are often found to have hits to reference protein families, or produce stable, high quality 3D models (Lin et al., 2022). As a result, families containing such sequences may actually represent protein fragments or protein domains that are either parts of larger, multi-domain sequences, or components of multimeric complexes.

Apart from the issues discussed above, which mostly pertain to the specifics of gene calling, an important drawback to the current metagenomic analysis workflows is their over-reliance on sequence homology-based annotation. Any sequences having no match to any reference databases are typically dropped from subsequent analysis in almost all metagenomic studies, which leaves the majority of the functional *dark matter* unexplored. Eliminating this need for reference datasets, can, in theory, be combated by performing all-vs-all analyses and annotation with novel approaches such as large scale clustering, deep learning-based structure prediction and synteny analysis. However, the above often require significant computational resources and scalability levels that are yet to be achieved.

Finally, a problem that needs to be addressed is the low quality, often incomplete metadata annotation for a large number of currently available metagenomic datasets, including ecosystem, geolocation and phylogeny associations. At the same time, different databases and repositories use different, often conflicting systems for assigning metadata to samples, leading to further confusion. The above, may ultimately result in poorly annotated contigs, MAGs and protein clusters. Some efforts have been made towards establishing a set of guidelines for annotating metagenomic samples (Kottmann et al., 2008; Vangay et al., 2021). However, unless these guidelines become a prerequisite for metagenomic data submission across multiple repositories, this issue will continue to exist.

## 9 Conclusion

In this review, we have presented and analyzed state-of-the-art, computational methods and approaches for analyzing metagenomic data at every step towards producing reliable protein clusters and annotating their function. Despite the limitations in the field, the recent developments have greatly expanded the available protein sequence space and provided novel tools for advances and innovations in biomedicine, biotechnology and ecology. Overall, we believe that this review can serve as a useful material and guidebook in the field of metagenomics, both for wet lab scientists and experienced bioinformaticians.

## Glossary

Microbiome: A community of microorganisms that can be found living together in any given habitat
Metagenome: The total amount of sequenced genetic material (DNA) from an environmental sample
Metatranscriptome: The total amount of actively expressed genes (RNA) from an environmental sample
Amplicon: A piece of DNA or RNA that is the source and product of amplification or replication events. It can be formed naturally through gene duplication, or artificially with polymerase chain reactions
Contig: A set of DNA segments or sequences that overlap in a way that provides a contiguous representation of a genomic region
Scaffold: A portion of a genome sequence reconstructed from end-sequenced whole-genome shotgun clones. Scaffolds are composed of contigs and gaps
Binning: The process of grouping reads or contigs into individual genomes and assigning each group to a specific taxon
Metagenome - assembled genome (MAG): A single-taxon assembly based on binned metagenomes that represents an entire individual genome
Paired-end shotgun sequencing: Also known as double-barrelled sequencing. Both ends of each fragment (5’ and 3′) are sequenced in order to make the process of reassembling the original target genome much faster, while also allowing for longer read lengths
Adapter sequences: Short oligonucleotides ligated to the ends of DNA fragments of interest, so that they can be combined with primers for amplification
Low-complexity regions: Sequence segments highly enriched in a single nucleotide/amino acid residue, or containing simple repeats (e.g., ATATATAT)
Sequence masking: The process of identifying and removing adapter sequences and low-complexity regions
Gene calling: The prediction of valid open reading frames (ORFs) for protein-coding genes in a sequence assembly
Non-coding RNAs (ncRNAs): Functional RNA molecules that are not translated into proteins. Examples include rRNAs, tRNAs, micro-RNAs *etc.*
CRISPR elements: A family of DNA sequences found in the genomes of prokaryotic organisms, derived from fragments of bacteriophages that had previously infected the prokaryote. They are used to detect and destroy similar bacteriophages during subsequent infections
Covariance Model (CM): Probabilistic model of the conserved sequence and secondary structure for an RNA family
Hidden Markov Model (HMM): A statistical Markov model in which the system being modeled is assumed to be a Markov process X) with unobservable (“hidden”) states, which influences an observable process Y) in a known way
Interpolated Markov Model (IMM): Variable-order Markov model, using a variable number of states to compute the probability of the next state
*k*-mers: Substrings of length *k* (e.g., 3-mers, 4-mers etc.) contained within a sequence
Deep Learning: A subset of AI and machine learning that uses multi-layered artificial neural networks to deliver state-of-the-art accuracy
Transformer module: A type of deep learning architecture, based primarily upon the self-attention module, designed for sequence-to-sequence tasks. Multiple transformer modules can be combined to process sequence information at various levels and derive its features (e.g., 3D structure).
